# The 1‐Year Functional Recovery From Severe COVID‐19 in a Swedish Working‐Age Cohort

**DOI:** 10.1111/aas.70058

**Published:** 2025-05-12

**Authors:** Björn Ahlström, Robert Frithiof, Michael Marks‐Hultström, Ing‐Marie Larsson, Gunnar Strandberg, Miklos Lipcsey

**Affiliations:** ^1^ Anesthesiology and Intensive Care, Department of Surgical Sciences Uppsala University Uppsala Sweden; ^2^ Centre for Clinical Research Dalarna Uppsala University Uppsala Sweden; ^3^ Integrative Physiology, Department of Medical Cell Biology Uppsala University Uppsala Sweden; ^4^ Hedenstierna Laboratory, Department of Surgical Sciences Uppsala University Uppsala Sweden

**Keywords:** critical care, functional recovery, hospital admission, recovery COVID‐19, sick leave

## Abstract

**Background:**

Long‐term symptoms are common after the acute phase of COVID‐19. We hypothesized that sick leave as an estimate of functional recovery, adjusted for confounding, differs between intensive care unit (ICU) and hospitalized COVID‐19 patients and population controls.

**Methods:**

In this cohort study, we identified all working‐age individuals with COVID‐19 admitted to ICUs or hospitals until July 20, 2020 from national registries. Matched population controls were randomly assigned to each ICU patient. Using logistic regression to adjust for confounding, we compared ICU patients to hospital patients and population controls on the number of sick leave‐free days alive during the first year after hospital discharge and the proportion of alive individuals on sick leave after 1 year.

**Results:**

We included 1020 COVID‐19 ICU patients, 5306 COVID‐19 hospital patients, and 4387 population controls. The ICU patients had a median of 271 (interquartile range, 33–349) sick leave‐free days alive, while hospital patients had 354 (334–365) and population controls 365 (365–365). ICU patients had an odds ratio (OR) of 0.14 (0.12–0.16, 95% confidence interval) compared to hospital patients, and 0.02 (0.02–0.03, both *p* < 0.001) compared to population controls for at least one more sick leave‐free day alive. Being on sick leave 1 year after inclusion had similar but inverse ORs.

**Conclusion:**

This national cohort study, in ICU and hospitalized patients with COVID‐19, shows that the severity of COVID‐19 disease, functional and health status before COVID‐19, and demographic factors had a major impact on recovery.

**Trial Registration:** NCT05054608

**Abbreviations**
CCI: Charlson comorbidity index
CI: confidence interval
COVID‐19: Corona virus disease 2019
ICD‐10: International Classification of Diseases—10th Revision
ICU: intensive care unit
IQR: interquartile range
NPR: National Patient Registry
OR: odds ratio
PIN: personal identification number
SIR: Swedish Intensive Care Registry
SSIA: Swedish Social Security Agency
TPR: Total Population Registry

## 1 Introduction

From early 2020, the Coronavirus disease 2019 (COVID‐19) burdened healthcare systems, especially intensive care units (ICUs). Short‐ and intermediate‐term mortality is high in hospital‐ and ICU‐admitted patients. However, mortality is low beyond 60–90 days from ICU admission [1, 2]. Apart from mortality, there are two distinct sets of consequences of ICU admission with COVID‐19: post‐COVID‐19 (also known as Long‐COVID) with multi‐organ impact, partially correlated to illness severity in non‐hospitalized individuals and post‐intensive care syndrome, related to multi‐organ failure and other factors during ICU stay [3, 4, 5, 6].

General long‐term consequences of COVID‐19 are well described [3]; however, long‐term consequences in persons admitted to ICU are less well documented, especially in large cohorts from national registries not exposed to selection bias from excluding the most severely affected individuals. Moreover, except for one large meta‐analysis reporting on Long‐COVID symptoms, but not the functional consequences [7], the existing cohort studies on health‐related quality of life and sick leave are burdened by small sample sizes or partially self‐reported data in contrast to the prospective nature and large size of national registry studies [8, 9, 10, 11, 12, 13, 14, 15, 16, 17, 18, 19], especially regarding ICU admitted patients. Return to work is a sensitive marker of recovered health in several disease categories, whereas inability to return to work is a strong marker of failed return of physical, cognitive, psychological, and social functions, that is, functional recovery [20, 21, 22, 23]. Return to work can be approximated by the end of sick leave, and the sickness cash benefit in Sweden is, by international standards, liberal and includes virtually the entire Swedish workforce but depends on medical assessment [24, 25]. Thus, the extent of not returning from sick leave is appropriate in approximating the return of function after severe illness based on medical assessment in a large cohort that covers the entire working population.

We hypothesized that ICU‐treated patients would have fewer sick leave‐free days alive after adjustment for relevant confounding, reflecting a more severely affected health and function than hospitalized COVID‐19 patients without ICU treatment or controls from the general population. We also expected a similar sick leave pattern 1 year after inclusion in 1‐year survivors. Accordingly, we conducted a nationwide registry‐based study of sick leave after severe COVID‐19.

## 2 Methods

This registry‐based cohort study with prospectively collected registry data is reported following the STROBE (strengthening the reporting of observational studies in epidemiology) guidelines [26]. The study was registered a priori on September 20, 2021, with a post hoc amendment on November 30, 2022, with ClinicalTrials.com (https://clinicaltrials.gov/study/NCT05054608).

### 2.1 Data Sources

The Swedish Intensive Care Registry (SIR), a level one quality registry, was used to identify ICU patients with COVID‐19. The registry covers all care episodes in all general ICUs in Sweden. The Swedish Board of Health and Welfare identified all individuals with at least one in‐patient episode with COVID‐19 in the National Patient Register (NPR). Reporting to the NPR is mandatory for all specialized care in Sweden. The NPR also provided information on previous health status. Statistics Sweden used the Total Population Registry (TPR) to draw random population control subjects, matched on age, sex, and area of residence, to the ICU patients in a 4:1 ratio. Statistics Sweden also provided demographic, labor market, and income information from the TPR and the Longitudinal integrated database for health insurance and labor market studies on all individuals in the study. These registries have complete coverage of all Swedish residents. Data on sick leave and sick pension, hereafter referred to as sick leave, were procured from the Swedish Social Insurance Agency (SSIA) which administers sickness cash benefits. Sickness cash benefits cover all individuals in the labor market; however, during the first 2 weeks of sick leave, the employer is responsible for sickness benefits. Thus, the agency has data on sick leave beyond the first 2 weeks of the sick leave period. Using the personal identification number (PIN) available for all Swedish residents, we could link data from the different registries.

### 2.2 Cohort

We compared three groups of individuals: the ICU group, the hospital group, and the population control group. The inclusion criteria included having a Swedish PIN, hospital discharge between January 1, 2020 and July 20, 2020, and age 18–63 years. The age interval captures working‐age adults at a 1‐year follow‐up. Exclusion criteria included having received pension the last year before admission or having extensive sick leave in the period 6 months to 2 weeks before admission. Extensive sick leave was defined as having either 28 days of continuous sick leave or having > 5 separate sick leave episodes during the period [27]. The ICU group included ICU patients discharged with a COVID‐19 diagnosis code (U07.1 in the International Classification of Diseases—10th Revision, ICD‐10) as identified in the SIR. The hospital group comprised hospital‐admitted patients, not admitted to the ICU, discharged with a COVID‐19 diagnosis code U07.1 identified in the NPR. Both registries demand a positive polymerase chain reaction test for Sars‐Cov‐2 to use the ICD‐10 code U07.1. The third group, the population control group, consisted of random individuals matched to the ICU cohort by sex, age, and county. The population controls were not admitted to hospital or ICU with COVID‐19. The hospital discharge date was used as the inclusion date for the ICU and hospital groups. The population controls had their matching ICU patient's discharge date as the inclusion date. The individuals were included on their first admission with COVID‐19.

### 2.3 Outcomes

Our primary outcome was odds ratio (OR) for having one or several more sick leave‐free days alive between the ICU patients and the hospital patients, as well as between the ICU patients and the population controls in ordinal logistic multiple regression. Sick leave‐free days alive was defined as the number of days the individual was alive without any degree of sick leave during the first year after inclusion. Our secondary outcome was OR for being on any degree of sick leave 1 year after inclusion between the ICU patients and the two control groups in binary logistic multiple regression models.

### 2.4 Statistics

Data are presented as medians with interquartile range (IQR) or numbers with percentages. Univariate comparisons were performed with the Mann–Whitney *U* test or chi‐square test as appropriate. Statistical significance was set at *p*‐value < 0.05 (two‐sided) and multiple comparisons in descriptive tables were addressed with the Bonferroni correction. Confidence intervals (CIs) were computed using a one‐sample binomial success rate for the proportion of individuals on sick leave per day after inclusion. For the primary outcome, we chose ordinal logistic regression with a logit link function to model sick leave‐free days alive based on Harrell's visual method [28]. Based on the estimate for the group variable, we calculated an unadjusted difference, with 95% CI in sick‐free days alive between groups using bootstrapping. Concordantly, we also estimated the adjusted difference between groups. The secondary outcome was modeled in binary logistic regression to assess the risk difference between groups for being on sick leave 1 year after inclusion. Lack of linearity between age and previous income and the outcomes was addressed by restricted cubic spline application in all models, and the ORs were calculated for the difference between the first and third quartiles [29]. The updated Charlson comorbidity index (CCI) was treated as a factor, and due to empty cells, we categorized CCI 6 to 11 into CCI 6 for the statistical models [30]. Finally, due to several zero values, we applied square root transformation to previous income for statistical modeling. However, despite this, the model on being on sick leave 1 year after inclusion did not converge for ICU patients and population controls until we excluded the previous income variable. Adjustment variables were chosen based on directed acyclic graphs. Definitions for model variables are listed in the Supporting Information (Table S1) and all variables in the models are displayed in the results figures with accompanying estimates. We did not perform conditional models on the matched strata, which would be inappropriate in this cohort study design [31].

### 2.5 Missing Data and Sensitivity Analyses

Missing data were imputed into 10 datasets and the regression model outputs were pooled [32]. The unadjusted and adjusted differences in sick‐free days alive between groups were calculated on complete cases. Several sensitivity analyses were performed and the rationale behind them is found in Table S2.

We used the R Software version 4.2.3 for data management, descriptive statistics and regression analyses, plots, and multiple imputations, (The R Foundation for Statistical Computing, Vienna, Austria; https://www.r‐project.org).

## 3 Results

We included 1020 patients in the ICU group, 5306 in the hospital group, and 4387 individuals in the population control group to analyze sick leave‐free days alive. For the analysis of the secondary outcome, being on sick leave 1 year after inclusion, individuals who died during follow‐up were excluded, leaving 880 patients in the ICU group, 5261 in the hospital group, and 4386 individuals in the population control group (Figure S1). The groups differed numerically in baseline characteristics (Table 1) and the baseline data for the cohort, in which 1‐year non‐survivors have been excluded, are given in Table S3.

**TABLE 1:** Baseline characteristics of ICU patients, hospital patients, and population controls.

	ICU patients	Hospital patients	Population controls
*N*	1020	5306	4387
Age	53 [46–58]	48 [38–56]	52 [45–58]
Sex, female	247 (24.2%)	2233 (42.1%)	1145 (26.1%)
CCI	0 (0–1)	0 (0–0)	0 (0–0)
SAPS3	49 (43–53)	—	—
Invasive mechanical ventilation	709 (69.5%)	—	—
Non‐invasive mechanical ventilation	204 (20.0%)	—	—
Continuous renal replacement therapy	132 (12.9%)	—	—
Surgical admission	20 (2.0)	—	—
Receiving unemployment benefit 1 year before inclusion	26 (1.9)	164 (2.4)	102 (1.8)
Annual income 1 year before inclusion, Euro	51,260 [33,660–67,540]	50,930 [31,570–67,540]	59,950 [44000–79,200]
Origin			
Born in Sweden to Swedish parents	388 (38.0%)	2168 (40.9%)	3177 (72.4%)
Born in a high‐income country or parents from a high‐income country outside Sweden	184(18.0%)	963 (18.1%)	586(13.4%)
Born in a low‐income country or parents from a low‐income country outside Sweden	448 (43.9%)	2175 (41.0%)	624 (14.2%)
Education			
Missing	28 (2.7%)	140 (2.6%)	71 (1.6%)
< 9 years elementary school	100 (10.1%)	497 (9.6%)	106 (2.5%)
9 years elementary school to < 3 years high school	369 (37.2%)	1754 (34.0%)	1574 (36.5%)
3 years high school to < 3 years in college/university	333 (33.6%)	1739 (33.7%)	1551 (35.9%)
≥ 3 years of college/university	190 (19.2%)	1176 (22.8%)	1085 (25.1%)
Civil status			
Missing	6 (0.6%)	26 (0.5%)	7(0.2%)
Married or in a registered partnership	571 (56.3%)	2825 (53.5%)	2160 (49.3%)
Unmarried	266 (26.2%)	1533 (29.0%)	1565 (35.7%)
Divorced	164 (16.2%)	873 (16.5%)	638 (14.6%)
Widowed	13 (1.3%)	49 (0.9%)	17 (0.4%)

*Note:* Data are presented as numbers with percentages or medians with [Q1–Q3]. Hospital patients were not admitted to an ICU. Population controls were not admitted to a hospital with COVID‐19. On December 31, 2019, 1 Swedish krona = 0.11 Euro [33].

Abbreviations: CCI, the updated Charlson comorbidity index; COVID‐19, coronavirus disease 2019; ICU, intensive care unit [30, 34]; SAPS3, Simplified Acute Physiology Score 3 [35].

### 3.1 Missing Data and Loss to Follow‐Up

Missing data were imputed in the highest education (2%) and civil status (0.4%) variables. Two individuals, one from the hospital group and one from the population control group, had an emigration date during the follow‐up period. These individuals were not excluded, but are effectively censored after emigration.

### 3.2 Crude Outcomes

**FIGURE 1:**
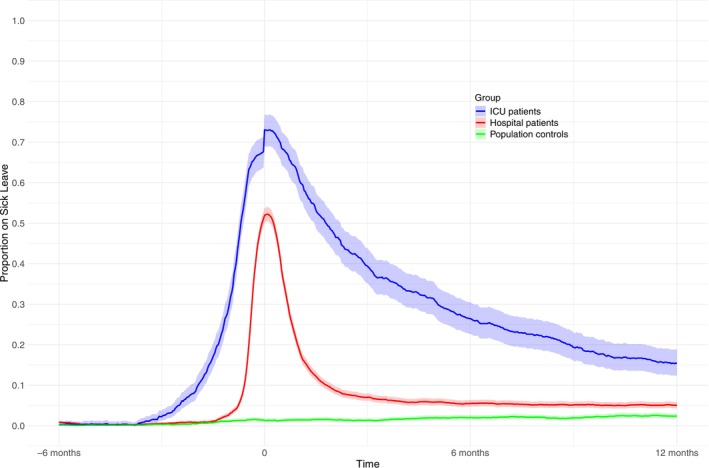
The proportion of alive individuals on sick leave, daily between 6 months before, to 1 year after inclusion, stratified by group. The gray area represents the 99% pointwise confidence interval. COVID‐19, coronavirus disease 2019; ICU, intensive care unit.

The number of sick leave‐free days alive was lowest in the ICU patients (median 271, 33–349, Q1–Q3), higher in the hospital patients (354, 334–365), and highest in the population controls (365, 365–365, Table 2). Using bootstrapping, we calculated the mean difference (with 95% CI) in sick leave‐free days alive. The ICU patients had 117 (108–126) fewer sick leave‐free days alive than the hospital patients and 146 (137–154) fewer than the population controls. The proportion of individuals on sick leave on each separate day from inclusion to day 365 was higher in the ICU group than in the hospital group and the population controls (Figure 1). In 15% (99% CI, 12–19) of ICU patients, 5% (4–6) of the hospital patients, and 2% (2–3) were on sick leave at day 365. The distribution of sick leave diagnoses is summarized in Figure S3. In Figure S3 we show the proportion of individuals on sick leave before exclusion of the individuals with extensive sick leave before inclusion.

**TABLE 2:** Outcomes 1 year after inclusion stratified by group.

	ICU patients	Hospital patients	*p*	Population controls	*p*
*N*	1020	5306		4387	
Sick leave‐free alive days first year after inclusion					
Median (IQR)	271 [33–349]	354 [334–365]	< 0.001	365 [365–365]	< 0.001
Mean (SD)	214 (145)	330 (71)		358 (34)	
Dead within 1 year	140 (13%)	45 (0.8%)	< 0.001	1 (0.02%)	< 0.001
Receiving pension after 1 year	13 (1.3%)	55 (1.0%)	> 0.99	86 (2.0%)	> 0.99
Receiving unemployment benefit after 1 year	28 (2.7%)	185 (3.5%)	> 0.99	101 (2.3%)	> 0.99
*n* (1‐year survivors)	880 (86%)	5261 (99%)		4386 (99%)	
On sick leave 1 year after inclusion	136 (15.5%)	263 (5.0%)	< 0.001	105 (2.4%)	< 0.001
Sick leave‐free days first year after inclusion					
Median (IQR)	303 [167–357]	354 [335–365]	< 0.001	365 [365–365]	< 0.001
Mean (SD)	248 (126)	333 (65)		358 (34)	
Receiving pension	13 (1.5%)	55 (1.0%)	> 0.99	86 (2.0%)	> 0.99
Receiving unemployment benefit	28 (3.2%)	185 (3.5%)	> 0.99	101 (2.3%)	> 0.99

*Note:* Data are presented as numbers with (percentages), medians with interquartile ranges [Q1–Q3] or mean with standard deviation (SD) as appropriate. Hospital patients were not admitted to an ICU. Population controls were not admitted to a hospital with COVID‐19. *p*‐values are Bonferroni transformed and refer to the difference between ICU patients and the column to the left of the *p*‐value.

Abbreviations: COVID‐19, coronavirus disease 2019; ICU, intensive care unit.

### 3.3 Logistic Models

**FIGURE 2:**
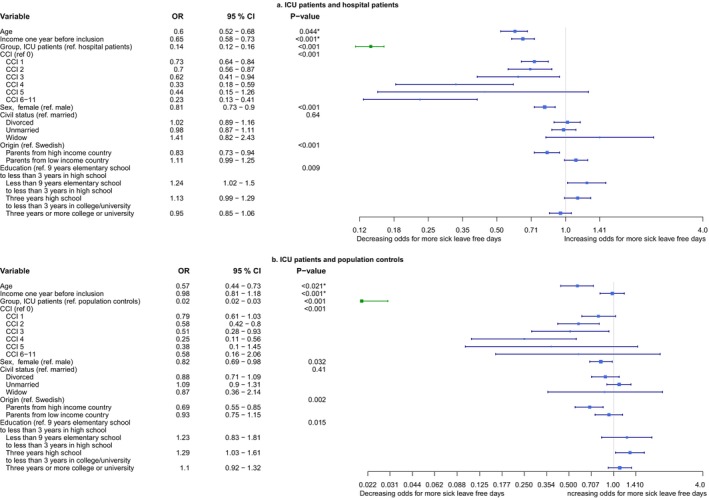
Ordinal logistic models on sick leave‐free days alive. (a) A forest plot of an ordinal logistic model on odds for having one or more additional sick leave‐free days alive during the first year after inclusion. ICU patients and hospital patients. (b) A forest plot of an ordinal logistic model on OR for having one or more additional sick leave‐free days alive during the first year after inclusion. ICU patients and population controls. *p‐value is for the non‐linear representation of the continuous variable, not the difference between quartiles. CCI updated Charlson comorbidity index; CI, confidence interval; COVID‐19, coronavirus disease 2019; ICU, intensive care unit; OR, odds ratio; NA, not applicable [30, 34].

Group affiliation was a significant variable in the ordinal logistic model on sick leave‐free days alive including ICU patients and hospital patients (OR 0.14, 0.12–0.16, 95% CI, *p* < 0.001) and in ICU patients and population controls (OR 0.02, 95% CI 0.02–0.03, *p* < 0.001). Thus, the ICU patients had lower odds of having another day sick free alive than the hospital patients and the population controls (Figure 2). We calculated the adjusted mean difference in sick leave‐free days alive from the models. The ICU group had 95 (85–106, 95% CI) less adjusted sick leave‐free days alive than the hospital patients and 136 (126–145) less than the population controls.

**FIGURE 3:**
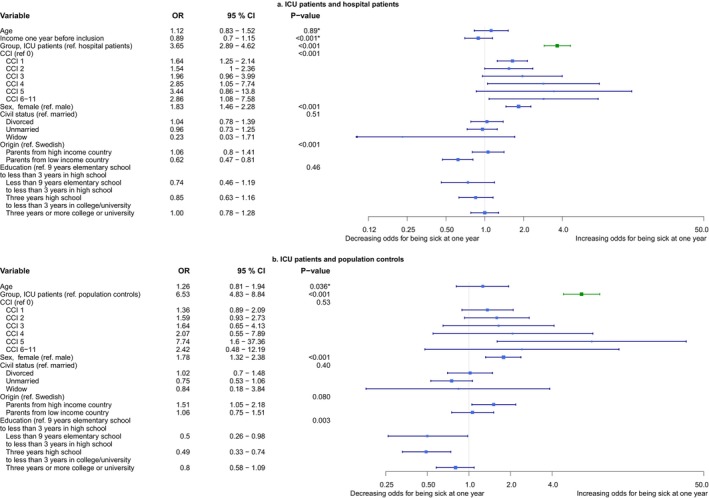
Binary logistic models on being on sick leave 1 year after inclusion. (a) A forest plot of a binary logistic model on odds for being on sick leave 1 year after inclusion. ICU patients and hospital patients. (b) A forest plot of a model on odds for being on sick leave 1 year after inclusion. ICU patients and population controls. *p‐value is for the non‐linear representation of the continuous variable, not the difference between quartiles. CCI updated Charlson comorbidity index; CI, confidence interval; COVID‐19, coronavirus disease 2019; ICU, intensive care unit; NA, not applicable; OR, odds ratio [30, 34].

In binary logistic models with being on sick leave 1 year after inclusion as outcome, performed on all individuals alive 1 year after inclusion, group affiliation was significant in ICU patients and hospital patients (OR 3.65, 95% CI 2.89–4.62, *p* < 0.001) and in ICU patients and population controls (OR 6.53, 95% CI 4.83–8.84, *p* < 0.001) (Figure 3).

### 3.4 Sensitivity Analyses

There were minor differences in the OR for the group variable between the primary and sensitivity analyses. These differences are summarized in Table S4. The detailed model results are provided in Tables S5–S20.

## 4 Discussion

In this nationwide cohort study, operationalizing functional recovery as sick leave‐free days alive, we found a slow and incomplete functional recovery in adjusted statistical models where patients with COVID‐19 admitted to the ICU were compared to hospitalized patients with COVID‐19 and population controls. The adjusted mean difference in sick leave‐free days alive between ICU patients and hospitalized patients was over 95 days, and the difference between ICU patients and population control individuals was 136 days. Thus, ICU patients are more exposed to sick leave than hospital patients and population controls, reflecting a slower, or incomplete functional recovery [9, 10, 11].

Sick‐leave benefit in Sweden is linked to repeated medical assessments of functional level and thereby can be used for assessing recovery after COVID‐19. Physicians assessing patients for sick‐leave benefit use decision support, usually integrated in the electronic patient records, administered by the Swedish Board of Health and Welfare [25]. Subsequently, the SSIA decides whether the patient has a severe enough functional impairment from medical reasons, based on the written opinion of the physician. Previous small cohort studies have reported a wide range of proportions of sick leave and return to work during the first year after hospital or ICU admission in patients with COVID‐19 disease. In hospitalized cohorts, in which varying proportions of patients were admitted to an ICU, return to work after 2–24 months varied from 41% to 89% [12, 13, 14, 36] and in ICU‐admitted patients, return to work was 51%–88% after 2–12 months [9, 10, 15, 16, 17, 18]. The latter outcomes contrast with our findings in that we report lower proportions being on sick leave. Returning to work, however, is a different endpoint than being on sick leave, as it implies that the individual is working at the time of inclusion. Moreover, returning to work is usually equivalent to returning to work to any extent, and our outcome is being on sick leave to any extent. Furthermore, we included working‐age individuals and excluded individuals who might not participate actively in the workforce, that is, patients who had received pension or had a high burden of sick leave in the period preceding the inclusion. Apart from the difference mentioned above in return to work and sick leave outcomes, there are two main reasons for different levels of sick leave between studies: (1) a registry‐based study like ours is not burdened with the lower follow‐up rate in the sickest patients seen in cohort studies with individual follow‐up; (2) the social security systems differ on sick leave. The Swedish healthcare system is generous, rendering sick leave a viable option for patients with remaining functional impairment. Based on the ordinal logistic models, we assessed the mean difference in sick leave‐free days alive before and after adjustment for confounding, unraveling substantial confounding where the difference was overestimated in the crude numbers. Moreover, in two binary logistic models, we studied the risk of being on sick leave 1 year after inclusion among surviving individuals. The ICU patients, compared to both the hospital patients and the population controls, had considerably higher odds for this outcome, reflecting the crude differences observed and the findings on the ordinal models. As the outcome measures days alive, the burden of not attaining functional recovery has to be added to the existing burden of short‐term mortality in patients discharged from hospital or ICU. Also, these outcomes are added to the burden of invasive and painful procedures, especially in patients admitted to ICU.

This work has some limitations. We did not have control over the original data collection process, but we used high‐quality registries with very high to complete coverage of the Swedish ICUs, specialist care, and the Swedish population. The sickness certification process is influenced by factors such as the prescriber's experience, and setting [37]. It can be assumed that these influences are comparable across the groups. We did not have access to actual return to work for the included individuals. However, according to the SSIA, 96% of individuals return to work at the end of a sick leave period making sick leave‐free days a relevant surrogate for functional recovery [24]. Another limitation is that the general population controls had COVID‐19 at low, but increasing rates over the study period [38]. The actual infection levels in the population controls are impossible to know, given that the testing capacity during the initial wave was minimal for the general public. We excluded patients with extensive sick leave before inclusion because of the strong correlation between sick leave and future sick leave and ill health [39, 40]. By doing so we introduced some level of confounding by selection bias, however, it was deemed that this selection was necessary. Another source of selection bias is the inclusion at hospital discharge which selects patients with shorter, before those with longer, hospital length of stay. However, this was inevitable to address the bias from sick leave during the hospital stay. Finally, although the proportion of individuals on sick leave is not generalizable outside Sweden our findings on impeded recovery are generalizable.

Our study has several strengths. The use of governmental registries to which reporting is governed by law and a high‐ranking national ICU registry to capture the exposure makes it equally unlikely that our findings are caused by exposure or outcome misclassification. Moreover, although the decision to prescribe sick leave is up to the individual physician and thus possibly biased, physicians use support documents from the SSIA. Additionally, all sick leave certifications must be approved by case officers at the SSIA. Importantly, any potential bias is likely evenly distributed across the groups under study. Furthermore, we report low frequencies of missing data, and we could use a reliable method of imputing to avoid selection bias from the exclusion of participants from the logistic models. Additionally, we were able to control for the expected baseline differences in socioeconomic variables between the ICU patients, hospital patients, and population controls. Finally, although we acquired the data retrospectively, they were collected prospectively to the registries, which removes the risk of recall bias.

## 5 Conclusion

This national cohort study, in ICU and hospitalized patients with COVID‐19, showed that the severity of COVID‐19 disease, functional and health status before COVID‐19, and demographic factors had a major impact on recovery.

## Author Contributions

All authors contributed to the study conception and design. Data collection was performed by Björn Ahlström and Miklos Lipcsey. Data management and statistical analysis was performed by Björn Ahlström. All authors had access to the data. Interpretation of the results was performed by all authors. The first draft of the manuscript was written by Björn Ahlström and all authors commented on previous versions of the manuscript. All authors read and approved the final manuscript.

## Ethics Statement

This study was performed in line with the principles of the Declaration of Helsinki. Approval was granted by the Regional Ethics Committee of Uppsala (EPM2020‐02144, with subsequent revisions).

## Consent

Informed consent was waived by the Regional Ethics Committee of Uppsala due to the nature of this registry study.

## Conflicts of Interest

The authors declare no conflicts of interest.

## Supporting information


**Data S1.** Supporting Information.

## Data Availability

The data that support the findings of this study are available from the registry holders (the Swedish Intensive Care Registry, Statistics Sweden, the Swedish Board of Health and Welfare, and the Social Security Agency) but restrictions apply to the availability of these data, which were used under license for the current study, and so are not publicly available. Data are however available from the authors upon reasonable request and with permission of the Swedish Ethical Review Authority and Uppsala University under the limitations of the European Data Protection Act.

## References

[1] E. Zettersten , L. Engerström , M. Bell , et al., “Long‐Term Outcome After Intensive Care for COVID‐19: Differences Between Men and Women—A Nationwide Cohort Study,” Critical Care 25, no. 1 (2021): 86. 33632273 10.1186/s13054-021-03511-x PMC7906087

[2] E. Hägglöf , M. Bell , E. Zettersten , L. Engerström , and E. Larsson , “Long‐Term Survival After Intensive Care for COVID‐19: A Nationwide Cohort Study of More Than 8000 Patients,” Annals of Intensive Care 13, no. 1 (2023): 76. 37642833 10.1186/s13613-023-01156-3 PMC10465451

[3] P. Brodin , G. Casari , L. Townsend , et al., “Studying Severe Long COVID to Understand Post‐Infectious Disorders Beyond COVID‐19,” Nature Medicine 28, no. 5 (2022): 879–882. 10.1038/s41591-022-01766-7 35383311

[4] A.‐F. Rousseau , H. C. Prescott , S. J. Brett , et al., “Long‐Term Outcomes After Critical Illness: Recent Insights,” Critical Care 25, no. 1 (2021): 108. 33731201 10.1186/s13054-021-03535-3 PMC7968190

[5] K. Nanwani‐Nanwani , L. López‐Pérez , C. Giménez‐Esparza , et al., “Prevalence of Post‐Intensive Care Syndrome in Mechanically Ventilated Patients With COVID‐19,” Scientific Reports 12, no. 1 (2022): 7977. 35562379 10.1038/s41598-022-11929-8 PMC9105588

[6] J. B. Soriano , S. Murthy , J. C. Marshall , P. Relan , and J. V. Diaz , “A Clinical Case Definition of Post‐COVID‐19 Condition by a Delphi Consensus,” Lancet Infectious Diseases 22, no. 4 (2022): e102–e107. 34951953 10.1016/S1473-3099(21)00703-9 PMC8691845

[7] Collaborators GBoDLC , “Estimated Global Proportions of Individuals With Persistent Fatigue, Cognitive, and Respiratory Symptom Clusters Following Symptomatic COVID‐19 in 2020 and 2021,” JAMA 328, no. 16 (2022): 1604–1615, 10.1001/jama.2022.18931. 36215063 PMC9552043

[8] P. Halvorsen , M. Hultström , J. Hästbacka , et al., “Health‐Related Quality of Life After Surviving Intensive Care for COVID‐19: A Prospective Multicenter Cohort Study,” Scientific Reports 13, no. 1 (2023): 18035. 37865685 10.1038/s41598-023-45346-2 PMC10590404

[9] I. M. Larsson , M. Hultström , M. Lipcsey , R. Frithiof , S. Rubertsson , and E. Wallin , “Poor Long‐Term Recovery After Critical COVID‐19 During 12 Months Longitudinal Follow‐Up,” Intensive & Critical Care Nursing 74 (2022): 103311. 36028412 10.1016/j.iccn.2022.103311 PMC9376301

[10] L. Carenzo , F. Dalla Corte , R. W. Haines , et al., “Return to Work After Coronavirus Disease 2019 Acute Respiratory Distress Syndrome and Intensive Care Admission: Prospective, Case Series at 6 Months From Hospital Discharge,” Critical Care Medicine 49, no. 11 (2021): e1157–e1162, 10.1097/CCM.0000000000005096. 34048368 PMC8507591

[11] E. Pauley , T. M. Drake , D. M. Griffith , et al., “Recovery From Covid‐19 Critical Illness: A Secondary Analysis of the ISARIC4C CCP‐UK Cohort Study and the RECOVER Trial,” Journal of the Intensive Care Society 24, no. 2 (2023): 162–169, 10.1177/17511437211052226. 37255989 PMC10225805

[12] O. Hürlimann , P. Decavel , J. M. Annoni , and M. Mancinetti , “Return to Work After Hospitalisation for COVID‐19 Infection,” European Journal of Internal Medicine 97 (2022): 110–112. 35031214 10.1016/j.ejim.2022.01.010 PMC8743451

[13] J. A. Frontera , D. Yang , A. Lewis , et al., “A Prospective Study of Long‐Term Outcomes Among Hospitalized COVID‐19 Patients With and Without Neurological Complications,” Journal of the Neurological Sciences 426 (2021): 117486. 34000678 10.1016/j.jns.2021.117486 PMC8113108

[14] L. Huang , Q. Yao , X. Gu , et al., “1‐Year Outcomes in Hospital Survivors With COVID‐19: A Longitudinal Cohort Study,” Lancet 398, no. 10302 (2021): 747–758. 34454673 10.1016/S0140-6736(21)01755-4 PMC8389999

[15] G. Monti , C. Leggieri , E. Fominskiy , et al., “Two‐Months Quality of Life of COVID‐19 Invasively Ventilated Survivors; an Italian Single‐Center Study,” Acta Anaesthesiologica Scandinavica 65, no. 7 (2021): 912–920. 33655487 10.1111/aas.13812 PMC8014684

[16] C. L. Hodgson , A. M. Higgins , M. J. Bailey , et al., “The Impact of COVID‐19 Critical Illness on New Disability, Functional Outcomes and Return to Work at 6 Months: A Prospective Cohort Study,” Critical Care 25, no. 1 (2021): 382. 34749756 10.1186/s13054-021-03794-0 PMC8575157

[17] N. van Veenendaal , I. C. van der Meulen , M. Onrust , W. Paans , W. Dieperink , and P. H. J. van der Voort , “Six‐Month Outcomes in COVID‐19 ICU Patients and Their Family Members: A Prospective Cohort Study,” Healthcare 9, no. 7 (2021): 865, 10.3390/healthcare9070865. 34356243 PMC8305246

[18] N. Latronico , E. Peli , S. Calza , et al., “Physical, Cognitive and Mental Health Outcomes in 1‐Year Survivors of COVID‐19‐Associated ARDS,” Thorax 77, no. 3 (2022): 300–303. 34588274 10.1136/thoraxjnl-2021-218064

[19] N. V. Skei , K. Moe , T. I. L. Nilsen , et al., “Return to Work After Hospitalization for Sepsis: A Nationwide, Registry‐Based Cohort Study,” Critical Care 27, no. 1 (2023): 443. 37968648 10.1186/s13054-023-04737-7 PMC10652599

[20] R. P. Dreyer , X. Xu , W. Zhang , et al., “Return to Work After Acute Myocardial Infarction,” Circulation. Cardiovascular Quality and Outcomes 9, no. 2 (2016): S45–S52. 26908859 10.1161/CIRCOUTCOMES.115.002611 PMC4771977

[21] H. de Vries , A. Fishta , B. Weikert , A. Rodriguez Sanchez , and U. Wegewitz , “Determinants of Sickness Absence and Return to Work Among Employees With Common Mental Disorders: A Scoping Review,” Journal of Occupational Rehabilitation 28, no. 3 (2018): 393–417. 28980107 10.1007/s10926-017-9730-1 PMC6096498

[22] L. M. Bek , J. C. Berentschot , M. E. Hellemons , et al., “Return to Work and Health‐Related Quality of Life up to 1 Year in Patients Hospitalized for COVID‐19: The CO‐FLOW Study,” BMC Medicine 21, no. 1 (2023): 380. 37784149 10.1186/s12916-023-03083-3 PMC10546751

[23] J. Selander , J. Sun , A. Tjulin , and N. Buys , “Interrelated Factors for Return to Work of Sick‐Listed Employees in Sweden,” International Journal of Disability Management 15 (2020): e7.

[24] Swedish Social Security Agency , “Lång väg tillbaka till arbete vid sjukskrivning [Long Way Back to Work From Sick Leave],” 2017, https://www.forsakringskassan.se/download/18.3a5418591814e228e4413ac/1661265485367/psykiatriska‐diagnoser‐korta‐analyser‐2017‐1.pdf.

[25] Försäkringsmedicinskt beslutsstöd , Medical Decision Support for Insurance Purposes (Swedish Board of Health and Welfare, 2024), https://www.socialstyrelsen.se/statistik‐och‐data/oppna‐data/forsakringsmedicinskt‐beslutsstod.

[26] E. von Elm , D. G. Altman , M. Egger , et al., “The Strengthening the Reporting of Observational Studies in Epidemiology (STROBE) Statement: Guidelines for Reporting Observational Studies,” Epidemiology 18, no. 6 (2007): 800–804. 18049194 10.1097/EDE.0b013e3181577654

[27] E. Westerlind , A. Palstam , K. S. Sunnerhagen , and H. C. Persson , “Patterns and Predictors of Sick Leave After Covid‐19 and Long Covid in a National Swedish Cohort,” BMC Public Health 21, no. 1 (2021): 1023. 34059034 10.1186/s12889-021-11013-2 PMC8164957

[28] F. E. Harrell , “Regression Models for Continuous Y and Case Study in Ordinal Regression,” in Regression Modeling Strategies. Springer Series in Statistics (Springer International Publishing, 2015), 359–387.

[29] F. E. Harrell , “Relaxing Linearity Assumption for Continuos Predictors,” in Regresion Modeling Strategies. Springer Series in Statistics (Springer International Publishing, 2015), 18–29.

[30] H. Quan , B. Li , C. M. Couris , et al., “Updating and Validating the Charlson Comorbidity Index and Score for Risk Adjustment in Hospital Discharge Abstracts Using Data From 6 Countries,” American Journal of Epidemiology 173, no. 6 (2011): 676–682. 21330339 10.1093/aje/kwq433

[31] T. L. Lash , T. J. VanderWeele , S. Haneuse , and K. J. Rothman , “Epidemiologic Study Design,” in Modern Epidemiology, 4th ed. (Wolters Kluwer, 2021), 105–140.

[32] S. Van Buuren , Multiple imputation. Flexible Inputation of Missing Data. Chapman & Hall/CRC Interdisciplinary Statistics (Chapman and Hall/CRC, 2018), 29–62.

[33] MBH Media Inc , “World Currencey Exchange Rates and Currency Exchange Rate History,” https://www.exchange‐rates.org/Rate/SEK/USD/12‐31‐2019.

[34] M. E. Charlson , P. Pompei , K. L. Ales , and C. R. MacKenzie , “A New Method of Classifying Prognostic Comorbidity in Longitudinal Studies: Development and Validation,” Journal of Chronic Diseases 40, no. 5 (1987): 373–383, 10.1016/0021-9681(87)90171-8. 3558716

[35] R. P. Moreno , P. G. Metnitz , E. Almeida , et al., “SAPS 3—From Evaluation of the Patient to Evaluation of the Intensive Care Unit. Part 2: Development of a Prognostic Model for Hospital Mortality at ICU Admission,” Intensive Care Medicine 31, no. 10 (2005): 1345–1355, 10.1007/s00134-005-2763-5. 16132892 PMC1315315

[36] L. Huang , X. Li , X. Gu , et al., “Health Outcomes in People 2 Years After Surviving Hospitalisation With COVID‐19: A Longitudinal Cohort Study,” Lancet Respiratory Medicine 10, no. 9 (2022): 863–876. 35568052 10.1016/S2213-2600(22)00126-6 PMC9094732

[37] K. Starzmann , P. Hjerpe , S. Dalemo , H. Ohlsson , C. Björkelund , and K. Bengtsson Boström , “Diagnoses Have the Greatest Impact on Variation in Sick‐Leave Certification Rate Among Primary‐Care Patients in Sweden: A Multilevel Analysis Including Patient, Physician and Primary Health‐Care Centre Levels,” Scandinavian Journal of Public Health 43, no. 7 (2015): 704–712, 10.1177/1403494815591898. 26122466

[38] Public Health Agency of Sweden , “Covid‐19—Statistics With Figures and Maps,” 2022, https://experience.arcgis.com/experience/09f821667ce64bf7be6f9f87457ed9aa.

[39] H. Hultin , C. Lindholm , M. Malfert , and J. Möller , “Short‐Term Sick Leave and Future Risk of Sickness Absence and Unemployment—The Impact of Health Status,” BMC Public Health 12, no. 1 (2012): 861. 23050983 10.1186/1471-2458-12-861 PMC3508966

[40] P. M. Dekkers‐Sánchez , J. L. Hoving , J. K. Sluiter , and M. H. W. Frings‐Dresen , “Factors Associated With Long‐Term Sick Leave in Sick‐Listed Employees: A Systematic Review,” Occupational and Environmental Medicine 65, no. 3 (2008): 153–157. 17881466 10.1136/oem.2007.034983

