# Supplementary material for: The 1‐Year Functional Recovery From Severe COVID‐19 in a Swedish Working‐Age Cohort
Source: Acta Anaesthesiol Scand. 2025 May 12;69(6):e70058. doi: 10.1111/aas.70058 (PMC12069831; doi:10.1111/aas.70058)
Supplement: Supplementary file 1 — Data S1. Supporting Information. [file AAS-69-0-s001.docx]

# **The one-year functional recovery from severe COVID-19 in a Swedish working-age cohort**

# **Online resource, Acta Anaesthesiologica Scandinavica**

*Björn Ahlström^1,2^, Robert Frithiof^1^, Michael Marks-Hultström^1,3^, Ing-Marie Larsson^1^, Gunnar Strandberg^1^, Miklos Lipcsey^1,4^

^1^Anesthesiology and Intensive Care, Department of Surgical Sciences, Uppsala University, Uppsala, Sweden. ^2^Centre for Clinical Research, Dalarna, Uppsala University, Sweden. ^3^Integrative Physiology, Department of Medical Cell Biology, Uppsala University, Uppsala, Sweden. ^4^Hedenstierna laboratory, Department of Surgical Sciences, Uppsala University, Uppsala, Sweden.

*Corresponding author

E-mail: [bjorn.ahlstrom@uu.se](mailto:bjorn.ahlstrom@uu.se)

**Table of contents**

[Table S1. Definition of covariates 3](#_Toc193280661)

[Table S2. Rational for sensitivity analyses. 4](#_Toc193280662)

[Figure S1. Patient selection flowchart. 5](#_Toc193280663)

[Figure S2. Proportion of alive individuals on sick leave, daily between 6 months before, to one year after inclusion stratified by group, before exclusion of individuals with extensive sick leave before inclusion. 6](#_Toc193280664)

[Figure S3. Distribution of diagnoses for sick leave in individuals on sick leave at one year after inclusion 7](#_Toc193280665)

[Table S3. Baseline characteristics 8](#_Toc193280666)

[Table S4. Summary of sensitivity analyses results. 9](#_Toc193280667)

[Table S5. Sensitivity analysis. 10](#_Toc193280668)

[Table S6. Sensitivity analysis. 11](#_Toc193280669)

[Table S7. Sensitivity analysis. 12](#_Toc193280670)

[Table S8. Sensitivity analysis. 13](#_Toc193280671)

[Table S9. Sensitivity analysis. 14](#_Toc193280672)

[Table S10. Sensitivity analysis. 15](#_Toc193280673)

[Table S11. Sensitivity analysis. 16](#_Toc193280674)

[Table S12. Sensitivity analysis. 17](#_Toc193280675)

[Table S13. Sensitivity analysis. 18](#_Toc193280676)

[Table S14. Sensitivity analysis. 19](#_Toc193280677)

[Table S15. Sensitivity analysis 20](#_Toc193280678)

[Table S16. Sensitivity analysis. 21](#_Toc193280679)

[Table S17. Sensitivity analysis. 22](#_Toc193280680)

[Table S18. Sensitivity analysis. 23](#_Toc193280681)

[Table S19. Sensitivity analysis. 24](#_Toc193280682)

[Table S20. Sensitivity analysis. 25](#_Toc193280683)

[References 26](#_Toc193280684)

| **Table S1. Definition of covariates** | |
| --- | --- |
| ICU patient | An ICU admitted patient with a discharge ICD-10 diagnosis U07.1 in the Swedish intensive care registry. All Swedish ICUs reported all ICU admissions during inclusion period. Patients <18 years and >63 years were excluded. |
| Hospital patient | A hospital admitted patient, not admitted to ICU with Covid-19 during the study period, with a discharge ICD-10 diagnosis U07.1 in the patient registry. Reporting of hospital admissions to the patient registry is mandatory by statutory and common law. Patients <18 years and >63 years were excluded. |
| Population control | A random individual from the Swedish Total population registry, not admitted to hospital with Covid-19 during the study period, matched to an ICU patient on age, legal gender and municipality by the population statistics. |
| Income one year before inclusion | Cumulative yearly income, in SEK, until one year before inclusion, not including social welfare, procured from the population statistics |
| Age | Age in years |
| CCI | The updated Charlson comorbidity index (1, 2) based on ICD-10 diagnoses in specialized care from 5 years preceding inclusion |
| Sex | The legal gender, procured from the population statistics |
| Civil status | The civil status, procured from the population statistics |
| Origin | All individuals were placed in an origin category based on their parents birthplace category, Sweden was prioritized before high income country which was prioritized before low income country.   \| Level \| Origin as reported from the Population registry \| \| --- \| --- \| \| Swedish \| Swedish \| \| High income country \| Europe, Oceania or North America \| \| Low income country \| Africa, Asia, South America, Unknown or Stateless \| |
| Education | Highest level of education, procured from the population statistics, completed level.   \| Level \| ISCED \| \| --- \| --- \| \| Less than 9 years elementary school \| X. No education or 0. Early childhood education \| \| 9 years in elementary school to less than 3 years in high school \| 1. Primary education or 2. Lower secondary education \| \| Three years high school or less than 3 years in college/university \| 3. Upper secondary education, 4. Post-secondary non-tertiary education or 5. Short-cycle tertiary education \| \| Three years or more college or university \| 6 Bachelor’s or equivalent level, or higher \| |
| On sick leave one year before inclusion | On sick leave or sick pension at any fraction on the day exactly one year *before* inclusion. Procured from the Swedish social insurance agency |
| *ICU* Intensive care unit *ICD-10* the International Classification of Diseases - tenth revision *SEK* Swedish kronor *ISCED* International Standard Classification of Education (3) | |

| **Table S2.** **Rationale for sensitivity analyses.** |
| --- |
| Several sensitivity analyses were performed. We repeated the regression models on complete cases to assess the imputation procedure. Moreover, we performed the binary logistic models with the outcome being on sick leave, unemployment benefit, or age pension one year after inclusion to assess the impact of alternate reasons to be off the labor market. The models on sick leave free days alive was not explored in this way because the data on unemployment and pension does not have the temporal resolution needed. The models were also preformed while restricting the ICU group to individuals subject to of invasive mechanical ventilation. In addition, we performed the models as Ordinary least squares linear regression because that is the intuitive model. Furthermore, to explore the effect of a previous mood or anxiety diagnosis, the most common cause of sick leave in Sweden, we added this to the ordinal and binary logistic models. Finally, we included individuals that originally were excluded due to extensive sick leave , and added the number of days on sick leave within 6 months to 2 weeks before admission. |

| 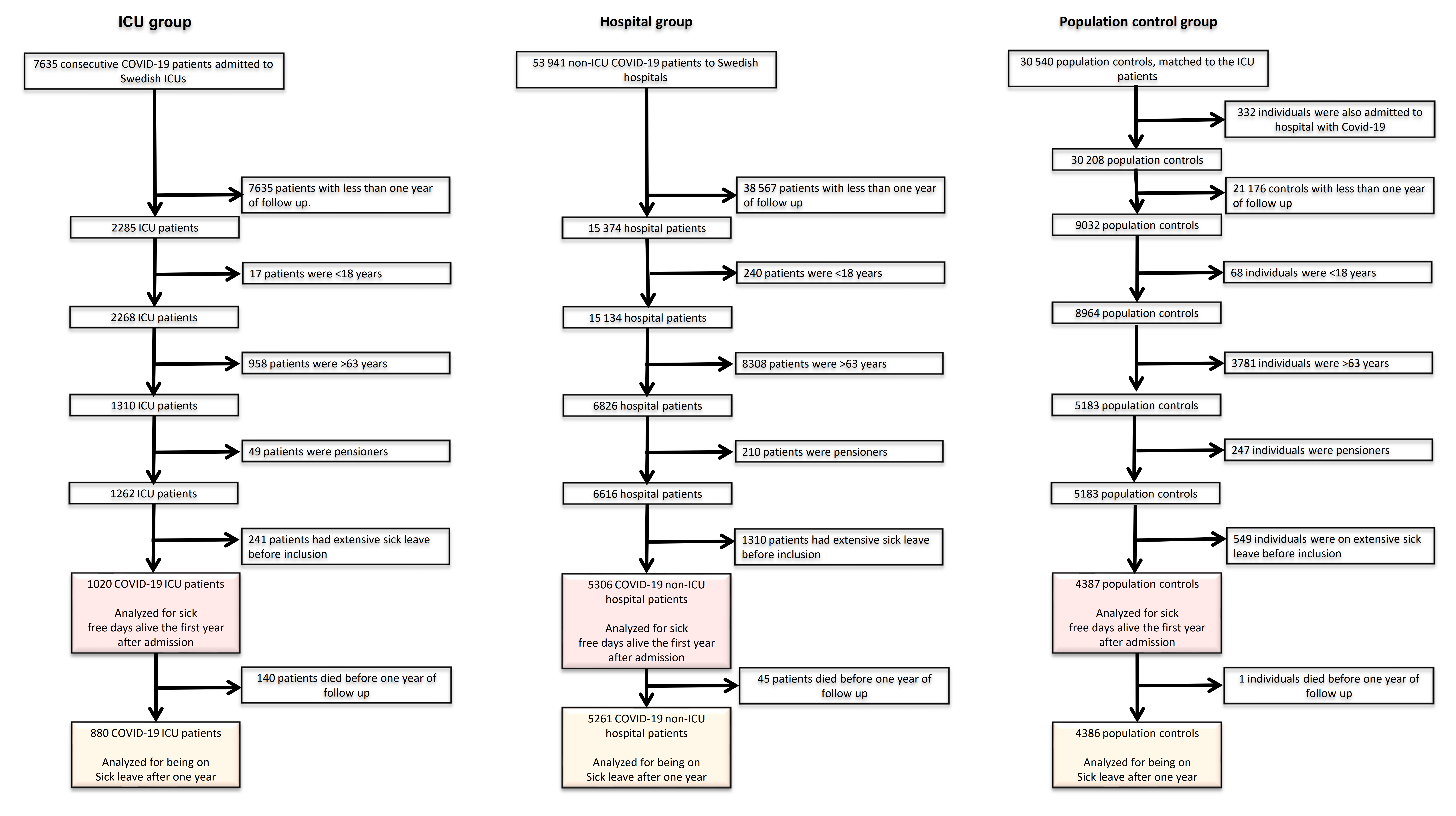 |
| --- |
| **Figure S1. Patient selection flowchart.**  *COVID-19* Coronavirus disease 2019 *ICU* Intensive care unit *PIN* Personal identification number. |

| 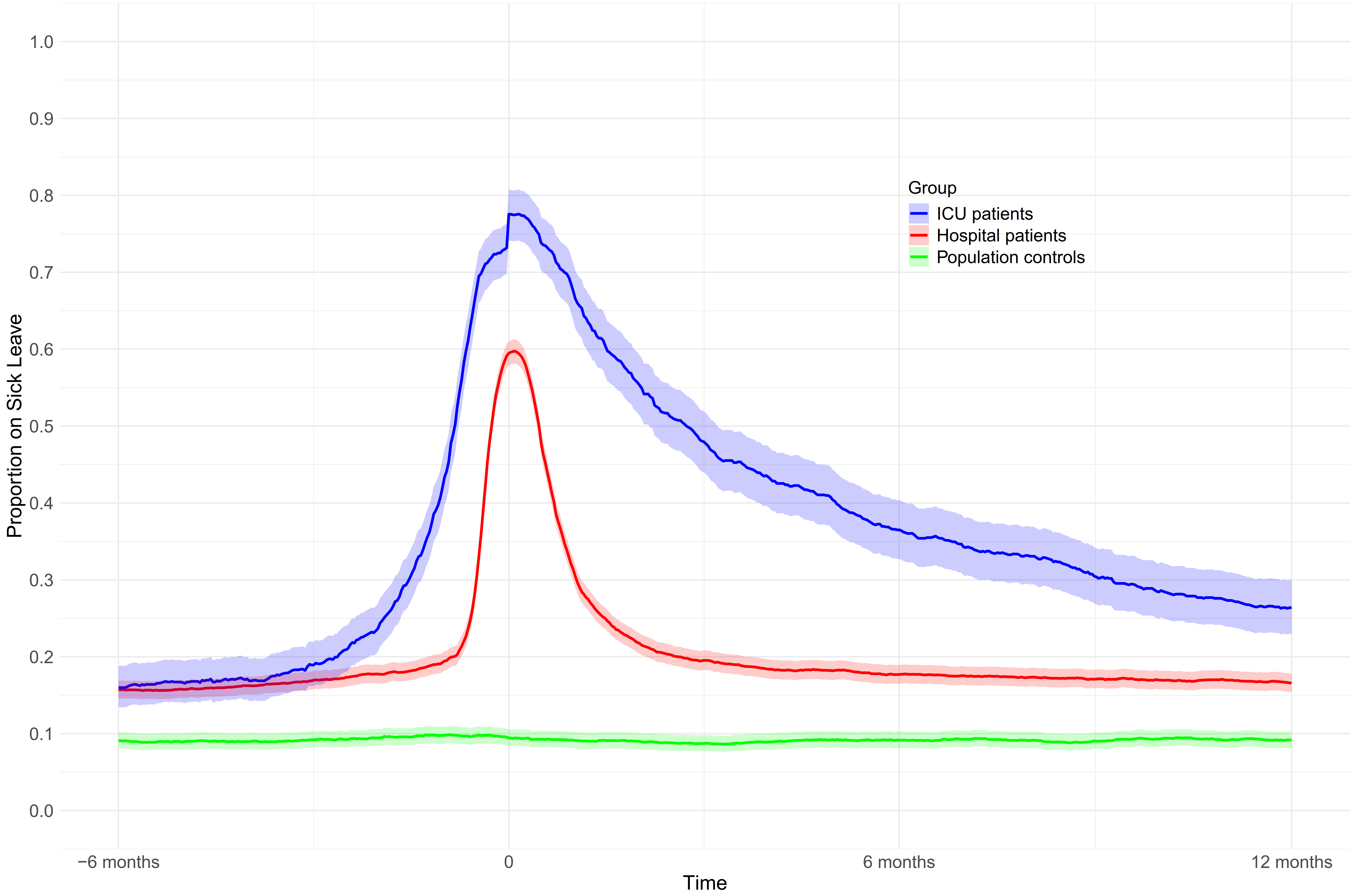 |
| --- |
| **Figure S2. The proportion of alive individuals on sick leave, daily between 6 months before, to one year after inclusion stratified by group, before exclusion of individuals with extensive sick leave before inclusion.**  The hued areas represent the 99% pointwise confidence interval. At day 365, ICU patients: 26% (99% CI, 23-30), hospital patients 17% (15-18) and population controls 9% (8-10). *COVID-19* Coronavirus disease 2019 *ICU* Intensive care unit. |

| 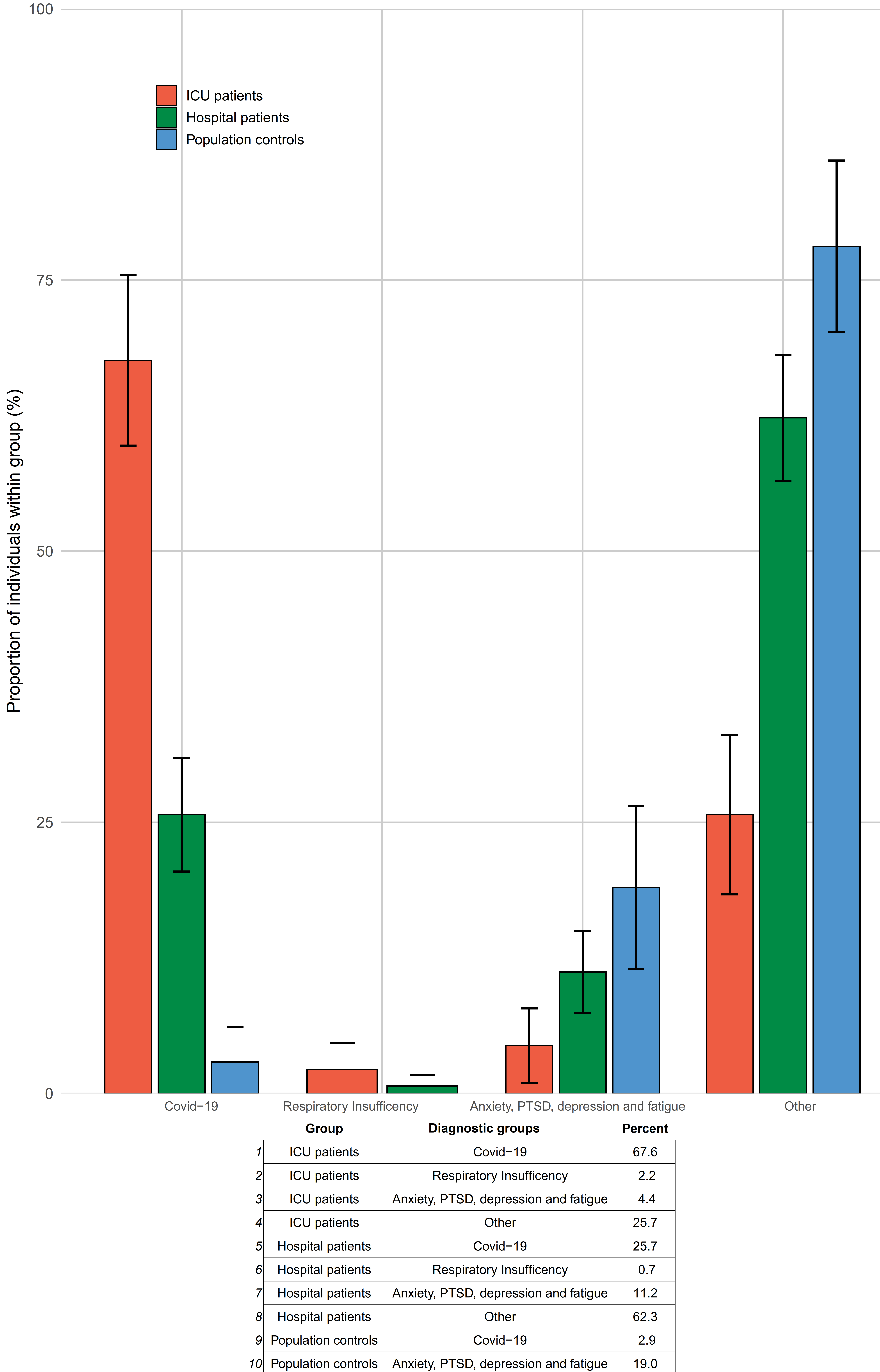 |
| --- |
| **Figure S3. Distribution of diagnoses for sick leave in individuals on sick leave at one year after inclusion.** ICU patients, hospital patients and population controls. Error bars and numbers within brackets represent crude sample 95 % confidence intervals. ICU patients are patients admitted to ICU with a discharge diagnosis of Covid-19, hospital patients are patients admitted to hospital, but not ICU, with a Covid-19 discharge diagnosis and population controls are control individuals not admitted to hospital with Covid-19 during study period. *Covid-19* Corona virus disease 2019 *ICU* Intensive care unit *PTSD* post-traumatic stress syndrome |
|  |

| **Table S3. Baseline characteristics of ICU patients, hospital patients and population analyzed for being on sick leave one year after inclusion.** Individuals deceased during observation are excluded. | | | |  |
| --- | --- | --- | --- | --- |
|  | ICU patients | Hospital patients | Population controls | |
| n | 880 | 5261 | 4386 | |
| Age | 52 (45-58) | 48.0 (38-56) | 52.0 (45-58) | |
| Sex, female | 221 (25.1%) | 2219 (42.2%) | 1145 (26.1%) | |
| CCI | 0 (0-1) | 0 (0-0) | 0 (0-0) | |
| SAPS3 | 49 (43-54) | -- | -- | |
| Invasive mechanical ventilation | 573 (65.1%) | -- | -- | |
| Non-invasive mechanical ventilation | 181 (20.6%) | -- | -- | |
| Continuous renal replacement therapy | 78 (8.9%)) | -- | -- | |
| Surgical admission | 28 (2.4) | -- | -- | |
| On sick leave 1 year before inclusion | 176 (14.7) | 957 (14.2) | 513 (9.2) | |
| Income 1 year before inclusion, EURO | 49 060 (26 180-66 000) | 48 290 (22 110-65 670) | 57 530 (38 280-76 780) | |
| Origin |  |  |  | |
| Born in Sweden to Swedish parents | 351 (39.9%) | 2153 (40.9%) | 3176 (72.4%) | |
| Born in a High income country or  parents from High income country  outside Sweden | 154 (17.5%) | 954 (18.1%) | 586 (13.4%) | |
| Born in a Low income country or  parents from Low income country  outside Sweden | 375 (42.6%) | 2154 (40.9%) | 624 (14.2%) | |
| Education |  |  |  | |
| Missing | 22 (2.5%) | 136 (2.6%) | 71 (1.6%) | |
| < 9 years elementary school | 79 (9.2%) | 490 (9.6%) | 106 (2.5%) | |
| 9 years in elementary school to < 3 years in  high school | 327 (38.1%) | 1733 (33.8%) | 1574 (36.5%) | |
| 3 years high school to < 3 years in  college/university | 286 (33.3%) | 1728 (33.7%) | 1550 (35.9%) | |
| ≥3 years college/university | 166 (19.3%) | 1174 (22.9%) | 1085 (25.1%) | |
| Civil status |  |  |  | |
| Missing | 6 (0.7%) | 25 (0.5%) | 7 (0.2%) | |
| Married or in registered partnership | 490 (56.1%) | 2802 (53.5%) | 2160 (49.3%) | |
| Unmarried | 237 (27.1%) | 1520 (29.0%) | 1565 (35.7%) | |
| Divorced | 137 (15.7%) | 867 (16.6%) | 637 (14.5%) | |
| Widowed | 10 (1.1%) | 47 (0.9%) | 17 (0.4%) | |
| Data are presented as numbers with percentages or medians with interquartile ranges as appropriate. ICU patients are patients admitted to ICU with a discharge diagnosis of Covid-19, hospital patients are patients admitted to hospital, but not ICU, with a Covid-19 discharge diagnosis and population controls are control individuals not admitted to hospital with Covid-19 during study period. There is no overlap between the groups. *Covid-19* Corona virus disease 2019 *ICU* Intensive care unit *CCI* the updated Charlson comorbidity index(1, 2) *SAPS3* Simplified Acute Physiology Score 3.(4) At 31^st^ December 2019, 1 Swedish krona = 0.11 EURO (5). | | | |  |

| **Table S4. Summary of sensitivity analysis results.** |
| --- |
| **Complete cases**  When we repeated the models on complete cases we found almost the same estimates for Group as in the main analyses (Tables S5 to S8, below).  **Widen the outcome to being on sick leave, unemployment or pension at one year after inclusion**  In the sensitivity analysis where the outcome was widened to being on sick leave, unemployment or pension at one year after inclusion The estimate for group was slightly lower than in the main analyses, but still highly significant (Tables S9 and S10, below).  **Restricting to ICU patients with invasive mechanical ventilation**  In the sensitivity analyses where only patients with invasive mechanical ventilation were included the estimates for Group were similar to those from the main analyses (Tables S11 to S14, below).  **Ordinary least squares linear regression**  In linear regression the association between group and the outcomes were very high as in the main, logistic, analyses (Tables S15 and S16, below).  **Mood and anxiety disorders**  When presence of comorbid mood or anxiety diagnoses were added to the ordinal and binary logistic models the estimates for Group were very similar to those of the main analyses (Tables S17 to S20).  **Extensive sick leave**  When we added individuals with preceding, extensive sick leave to the cohort and extended the models with the number of preceding sick days the impact from ICU care was slightly attenuated compared to the main analyses (Tables S21 to S24). |
| ICU patients are patients admitted to ICU with a discharge diagnosis of Covid-19, hospital patients are patients admitted to hospital, but not ICU, with a Covid-19 discharge diagnosis and population controls are control individuals not admitted to hospital with Covid-19 during study period. *Covid-19* Corona virus disease 2019 *ICU* Intensive care unit *CCI* the updated Charlson comorbidity index(1, 2) |

| **Table S5. Sensitivity analysis.** Ordinal logistic regression on odds ratio for having one or several more sick leave free days. *ICU patients and hospital patients.* Only complete cases included. | | | | |
| --- | --- | --- | --- | --- |
|  |  | 95% CI | |  |
|  | OR | Lower | Upper | *P*-value |
| Age | 0.60 | 0.52 | 0.69 | 0.028* |
| Income one year before inclusion | 0.65 | 0.58 | 0.73 | <0.001* |
| Group, ICU patients (ref: hospital patients) | 0.14 | 0.12 | 0.16 | <0.001 |
| CCI (ref 0) |  |  |  | <0.001 |
| CCI 1 | 0.74 | 0.65 | 0.85 |  |
| CCI 2 | 0.72 | 0.58 | 0.90 |  |
| CCI 3 | 0.63 | 0.42 | 0.96 |  |
| CCI 4 | 0.38 | 0.21 | 0.69 |  |
| CCI 5 | 0.61 | 0.21 | 1.76 |  |
| CCI 6-11 | 0.25 | 0.14 | 0.45 |  |
| Sex, female (ref: male) | 0.79 | 0.72 | 0.88 | <0.001 |
| Civil status (ref Married) |  |  |  | 0.52 |
| Civil status, Divorced | 1.02 | 0.9 | 1.16 |  |
| Civil status, Unmarried | 0.98 | 0.87 | 1.10 |  |
| Civil status, Widow | 1.50 | 0.85 | 2.64 |  |
| Origin (ref Swedish) |  |  |  | <0.001 |
| Origin, Parents from high income country | 0.81 | 0.71 | 0.93 |  |
| Origin, Parents from low income country | 1.09 | 0.97 | 1.23 |  |
| Education (ref: 9 years in elementary school to less than 3 years in high school) |  |  |  | 0.004 |
| Education, less than 9 years elementary school | 1.27 | 1.05 | 1.55 |  |
| Education, Three years high school or less than 3 years in college/university | 1.14 | 0.99 | 1.30 |  |
| Education, Three years or more college or university | 0.95 | 0.85 | 1.06 |  |
|  |  |  |  |  |
| ICU patients are patients admitted to ICU with a discharge diagnosis of Covid-19 and hospital patients are patients admitted to hospital, but not ICU, with a Covid-19 discharge diagnosis. *P-value is for the non-linear representation of the continuous variable, not the difference between quartiles. *Covid-19* Corona virus disease 2019 *OR* odds ratio *CI* Confidence interval, *ICU* Intensive care unit, *CCI* Updated Charlson comorbidity index(1, 2) | | | | |

| **Table S6. Sensitivity analysis.** Ordinal logistic regression on odds ratio for having one or several more sick leave free days. *ICU patients and population controls.* Only complete cases included. | | | | |
| --- | --- | --- | --- | --- |
|  |  | 95% CI | |  |
|  | OR | Lower | Upper | *P*-value |
| Age | 0.60 | 0.46 | 0.76 | 0.071* |
| Income one year before inclusion | 0.98 | 0.81 | 1.19 | <0.001* |
| Group, ICU patients (ref: population controls) | 0.02 | 0.02 | 0.03 | <0.001 |
| CCI (ref 0) |  |  |  | <0.001 |
| CCI 1 | 0.79 | 0.6 | 1.02 |  |
| CCI 2 | 0.61 | 0.44 | 0.84 |  |
| CCI 3 | 0.52 | 0.29 | 0.95 |  |
| CCI 4 | 0.26 | 0.11 | 0.6 |  |
| CCI 5 | 0.38 | 0.1 | 1.47 |  |
| CCI 6-11 | 0.59 | 0.16 | 2.11 |  |
| Sex, female (ref: male) | 0.83 | 0.69 | 0.99 | 0.040 |
| Civil status (ref Married) |  |  |  | 0.48 |
| Civil status, Divorced | 0.89 | 0.72 | 1.10 |  |
| Civil status, Unmarried | 1.09 | 0.9 | 1.31 |  |
| Civil status, Widow | 1.10 | 0.44 | 2.74 |  |
| Origin (ref Swedish) |  |  |  | 0.001 |
| Origin, Parents from high income country | 0.66 | 0.53 | 0.82 |  |
| Origin, Parents from low income country | 0.89 | 0.72 | 1.11 |  |
| Education (ref: 9 years in elementary school to less than 3 years in high school) |  |  |  | 0.11 |
| Education, less than 9 years elementary school | 1.28 | 0.86 | 1.89 |  |
| Education, Three years high school or less than 3 years in college/university | 1.31 | 1.04 | 1.63 |  |
| Education, Three years or more college or university | 1.10 | 0.92 | 1.32 |  |
|  |  |  |  |  |
| ICU patients are patients admitted to ICU with a discharge diagnosis of Covid-19 and population controls are control individuals not admitted to hospital with Covid-19 during study period. *P-value is for the non-linear representation of the continuous variable, not the difference between quartiles.*Covid-19* Corona virus disease 2019 *OR* Odds ratio *CI* Confidence interval, *ICU* Intensive care unit, *CCI* Updated Charlson comorbidity index(1, 2) | | | | |

| **Table S7. Sensitivity analysis.** Binary logistic regression on odds ratio for being on sick leave one year after inclusion. *ICU patients and hospital patients.* Only complete cases included. | | | | |
| --- | --- | --- | --- | --- |
|  |  | 95% CI | |  |
|  | OR | Lower | Upper | *P*-value |
| Age | 1.14 | 0.84 | 1.54 | 0.54* |
| Income one year before inclusion | 0.88 | 0.68 | 1.14 | <0.001* |
| Group, ICU patients (ref: hospital patients) | 3.71 | 2.93 | 4.69 | <0.001 |
| CCI (ref 0) |  |  |  | <0.001 |
| CCI 1 | 1.61 | 1.23 | 2.11 |  |
| CCI 2 | 1.53 | 1.00 | 2.35 |  |
| CCI 3 | 1.91 | 0.93 | 3.89 |  |
| CCI 4 | 2.93 | 1.07 | 7.98 |  |
| CCI 5 | 3.32 | 0.83 | 13.27 |  |
| CCI 6-11 | 2.79 | 1.05 | 7.4 |  |
| Sex, female (ref: male) | 1.84 | 1.47 | 2.3 | <0.001 |
| Civil status (ref Married) |  |  |  | 0.54 |
| Civil status, Divorced | 1.03 | 0.77 | 1.38 |  |
| Civil status, Unmarried | 0.96 | 0.74 | 1.26 |  |
| Civil status, Widow | 0.24 | 0.03 | 1.76 |  |
| Origin (ref Swedish) |  |  |  | <0.001 |
| Origin, Parents from high income country | 1.07 | 0.81 | 1.42 |  |
| Origin, Parents from low income country | 0.63 | 0.48 | 0.83 |  |
| Education (ref: 9 years in elementary school to less than 3 years in high school) |  |  |  | 0.32 |
| Education, less than 9 years elementary school | 0.72 | 0.45 | 1.15 |  |
| Education, Three years high school or less than 3 years in college/university | 0.85 | 0.63 | 1.16 |  |
| Education, Three years or more college or university | 1.00 | 0.78 | 1.28 |  |
|  |  |  |  |  |
| ICU patients are patients admitted to ICU with a discharge diagnosis of Covid-19 and hospital patients are patients admitted to hospital, but not ICU, with a Covid-19 discharge diagnosis. *P-value is for the non-linear representation of the continuous variable, not the difference between quartiles. *Covid-19* Corona virus disease 2019 *OR* Odds ratio *CI* Confidence interval, *ICU* Intensive care unit, *CCI* Updated Charlson comorbidity index(1, 2) | | | | |

| **Table S8. Sensitivity analysis.** Binary logistic regression on odds ratio for being on sick leave one year after inclusion. ICU patients and population controls. Only complete cases included. | | | | |
| --- | --- | --- | --- | --- |
|  |  | 95% CI | |  |
|  | OR | Lower | Upper | *P*-value |
| Age | 1.18 | 0.76 | 1.82 | 0.59* |
| Group, ICU patients (ref: population controls) | 6.60 | 4.88 | 8.93 | <0.001 |
| CCI (ref 0) |  |  |  | 0.66 |
| CCI 1 | 1.35 | 0.88 | 2.07 |  |
| CCI 2 | 1.58 | 0.92 | 2.71 |  |
| CCI 3 | 1.58 | 0.63 | 3.98 |  |
| CCI 4 | 2.22 | 0.57 | 8.63 |  |
| CCI 5 | 7.69 | 1.59 | 37.24 |  |
| CCI 6-11 | 2.31 | 0.45 | 11.78 |  |
| Sex, female (ref: male) | 1.81 | 1.35 | 2.43 | <0.001 |
| Civil status (ref Married) |  |  |  | 0.38 |
| Civil status, Divorced | 0.99 | 0.68 | 1.44 |  |
| Civil status, Unmarried | 0.73 | 0.52 | 1.04 |  |
| Civil status, Widow | 0.80 | 0.18 | 3.69 |  |
| Origin (ref Swedish) |  |  |  | 0.030 |
| Origin, Parents from high income country | 1.64 | 1.13 | 2.37 |  |
| Origin, Parents from low income country | 1.13 | 0.79 | 1.61 |  |
| Education (ref: 9 years in elementary school to less than 3 years in high school) |  |  |  | 0.002 |
| Education, less than 9 years elementary school | 0.48 | 0.25 | 0.94 |  |
| Education, Three years high school or less than 3 years in college/university | 0.48 | 0.32 | 0.71 |  |
| Education, Three years or more college or university | 0.78 | 0.57 | 1.07 |  |
|  |  |  |  |  |
| ICU patients are patients admitted to ICU with a discharge diagnosis of Covid-19 and population controls are control individuals not admitted to hospital with Covid-19 during study period. *P-value is for the non-linear representation of the continuous variable, not the difference between quartiles. *Covid-19* Corona virus disease 2019 *OR* Odds ratio *CI* Confidence interval, *ICU* Intensive care unit, *CCI* Updated Charlson comorbidity index(1, 2) | | | | |

| **Table S9. Sensitivity analysis.** Binary logistic regression on odds ratio for being on sick leave, unemployment or pension one year after inclusion. *ICU patients and hospital patients*. | | | | |
| --- | --- | --- | --- | --- |
|  |  | 95% CI | |  |
|  | OR | Lower | Upper | *P*-value |
| Age | 1.03 | 0.81 | 1.31 | <0.001* |
| Income one year before inclusion | 0.75 | 0.62 | 0.91 | <0.001* |
| Group, ICU patients (ref: hospital patients) | 2.40 | 1.97 | 2.93 | <0.001 |
| CCI (ref 0) |  |  |  | 0.15 |
| CCI 1 | 1.21 | 0.97 | 1.52 |  |
| CCI 2 | 1.12 | 0.77 | 1.62 |  |
| CCI 3 | 1.65 | 0.89 | 3.05 |  |
| CCI 4 | 1.7 | 0.68 | 4.24 |  |
| CCI 5 | 2.91 | 0.83 | 10.26 |  |
| CCI 6-11 | 1.64 | 0.67 | 4.01 |  |
| Sex, female (ref: male) | 1.44 | 1.21 | 1.72 | <0.001* |
| Civil status (ref Married) |  |  |  | 0.20 |
| Civil status, Divorced | 0.93 | 0.74 | 1.17 |  |
| Civil status, Unmarried | 0.87 | 0.7 | 1.08 |  |
| Civil status, Widow | 0.34 | 0.1 | 1.14 |  |
| Origin (ref Swedish) |  |  |  | 0.29 |
| Origin, Parents from high income country | 1.1 | 0.87 | 1.38 |  |
| Origin, Parents from low income country | 0.91 | 0.74 | 1.13 |  |
| Education (ref: 9 years in elementary school to less than 3 years in high school) |  |  |  | 0.13 |
| Education, less than 9 years elementary school | 1.12 | 0.82 | 1.52 |  |
| Education, Three years high school or less than 3 years in college/university | 0.81 | 0.63 | 1.03 |  |
| Education, Three years or more college or university | 0.85 | 0.70 | 1.04 |  |
|  |  |  |  |  |
| ICU patients are patients admitted to ICU with a discharge diagnosis of Covid-19 and hospital patients are patients admitted to hospital, but not ICU, with a Covid-19 discharge diagnosis. *P-value is for the non-linear representation of the continuous variable, not the difference between quartiles. *Covid-19* Corona virus disease 2019 *OR* Odds ratio *CI* Confidence interval, *ICU* Intensive care unit, *CCI* Updated Charlson comorbidity index(1, 2) | | | | |

| **Table S10. Sensitivity analysis.** Binary logistic regression on odds ratio for being on sick leave, unemployment or pension one year after inclusion. ICU patients and population controls. | | | | |
| --- | --- | --- | --- | --- |
|  |  | 95% CI | |  |
|  | OR | Lower | Upper | *P*-value |
| Age | 0.97 | 0.70 | 1.35 | <0.001* |
| Group, ICU patients (ref: population controls) | 2.97 | 2.35 | 3.76 | <0.001 |
| CCI (ref 0) |  |  |  | 0.016 |
| CCI 1 | 1.38 | 0.96 | 1.98 |  |
| CCI 2 | 1.56 | 1.03 | 2.36 |  |
| CCI 3 | 1.39 | 0.61 | 3.13 |  |
| CCI 4 | 2.62 | 0.88 | 7.86 |  |
| CCI 5 | 6.68 | 1.6 | 27.83 |  |
| CCI 6-11 | 1.24 | 0.25 | 6.22 |  |
| Sex, female (ref: male) | 1.35 | 1.08 | 1.68 | 0.009 |
| Civil status (ref Married) |  |  |  | 0.41 |
| Civil status, Divorced | 1.09 | 0.83 | 1.43 |  |
| Civil status, Unmarried | 0.87 | 0.68 | 1.11 |  |
| Civil status, Widow | 0.57 | 0.16 | 2.03 |  |
| Origin (ref Swedish) |  |  |  | 0.020 |
| Origin, Parents from high income country | 1.35 | 1.02 | 1.8 |  |
| Origin, Parents from low income country | 1.39 | 1.06 | 1.8 |  |
| Education (ref: 9 years in elementary school to less than 3 years in high school) |  |  |  | <0.001 |
| Education, less than 9 years elementary school | 0.57 | 0.34 | 0.95 |  |
| Education, Three years high school or less than 3 years in college/university | 0.52 | 0.39 | 0.7 |  |
| Education, Three years or more college or university | 0.76 | 0.6 | 0.96 |  |
|  |  |  |  |  |
| ICU patients are patients admitted to ICU with a discharge diagnosis of Covid-19 and population controls are control individuals not admitted to hospital with Covid-19 during study period. *P-value is for the non-linear representation of the continuous variable, not the difference between quartiles. *Covid-19* Corona virus disease 2019 *OR* Odds ratio *CI* Confidence interval, *ICU* Intensive care unit, *CCI* Updated Charlson comorbidity index(1, 2) | | | | |

| **Table S11. Sensitivity analysis.** Ordinal logistic regression on odds ratio for having one or several more sick leave free days. *ICU patients and hospital patients.* Only ICU patients with invasive mechanical ventilation. | | | | |
| --- | --- | --- | --- | --- |
|  |  | 95% CI | |  |
|  | OR | Lower | Upper | *P*-value |
| Age | 0.60 | 0.53 | 0.69 | 0.034* |
| Income one year before inclusion | 0.67 | 0.6 | 0.76 | <0.001* |
| Group, ICU patients (ref: hospital patients) | 0.08 | 0.07 | 0.09 | <0.001 |
| CCI (ref 0) |  |  |  | <0.001 |
| CCI 1 | 0.75 | 0.65 | 0.86 |  |
| CCI 2 | 0.7 | 0.56 | 0.88 |  |
| CCI 3 | 0.56 | 0.36 | 0.86 |  |
| CCI 4 | 0.34 | 0.19 | 0.63 |  |
| CCI 5 | 0.48 | 0.16 | 1.45 |  |
| CCI 6-11 | 0.19 | 0.11 | 0.35 |  |
| Sex, female (ref: male) | 0.8 | 0.73 | 0.89 | <0.001 |
| Civil status (ref Married) |  |  |  | 0.75 |
| Civil status, Divorced | 1.02 | 0.9 | 1.17 |  |
| Civil status, Unmarried | 1.00 | 0.88 | 1.13 |  |
| Civil status, Widow | 1.37 | 0.77 | 2.44 |  |
| Origin (ref Swedish) |  |  |  | 0.001 |
| Origin, Parents from high income country | 0.86 | 0.75 | 0.98 |  |
| Origin, Parents from low income country | 1.12 | 0.99 | 1.26 |  |
| Education (ref: 9 years in elementary school to less than 3 years in high school) |  |  |  | 0.009 |
| Education, less than 9 years elementary school | 1.27 | 1.04 | 1.55 |  |
| Education, Three years high school or less than 3 years in college/university | 1.12 | 0.98 | 1.29 |  |
| Education, Three years or more college or university | 0.95 | 0.85 | 1.07 |  |
|  |  |  |  |  |
| ICU patients are patients admitted to ICU with a discharge diagnosis of Covid-19 and hospital patients are patients admitted to hospital, but not ICU, with a Covid-19 discharge. *P-value is for the non-linear representation of the continuous variable, not the difference between quartiles. *Covid-19* Corona virus disease 2019 *OR* Odds ratio *CI* Confidence interval, *ICU* Intensive care unit, *CCI* Updated Charlson comorbidity index(1, 2) | | | | |

| **Table S12. Sensitivity analysis.** Ordinal logistic regression on odds ratio for having one or several more sick leave free days*. Covid-19 ICU patients and population controls.* Only ICU patients with invasive mechanical ventilation. | | | | |
| --- | --- | --- | --- | --- |
|  |  | 95% CI | |  |
|  | OR | Lower | Upper | *P*-value |
| Age | 0.57 | 0.44 | 0.75 | 0.019* |
| Income one year before inclusion | 1.11 | 0.9 | 1.37 | <0.001* |
| Group, ICU patients (ref: population controls) | 0.02 | 0.01 | 0.02 | <0.001 |
| CCI (ref 0) |  |  |  | <0.001 |
| CCI 1 | 0.87 | 0.64 | 1.19 |  |
| CCI 2 | 0.59 | 0.42 | 0.84 |  |
| CCI 3 | 0.39 | 0.2 | 0.77 |  |
| CCI 4 | 0.28 | 0.12 | 0.64 |  |
| CCI 5 | 0.42 | 0.09 | 1.92 |  |
| CCI 6-11 | 0.36 | 0.1 | 1.33 |  |
| Sex, female (ref: male) | 0.81 | 0.67 | 0.98 | 0.034 |
| Civil status (ref Married) |  |  |  | 0.19 |
| Civil status, Divorced | 0.86 | 0.68 | 1.09 |  |
| Civil status, Unmarried | 1.13 | 0.93 | 1.39 |  |
| Civil status, Widow | 0.64 | 0.23 | 1.83 |  |
| Origin (ref Swedish) |  |  |  | 0.026 |
| Origin, Parents from high income country | 0.73 | 0.57 | 2.14 |  |
| Origin, Parents from low income country | 0.89 | 0.7 | 1.66 |  |
| Education (ref: 9 years in elementary school to less than 3 years in high school) |  |  | 1.39 | 0.13 |
| Education, less than 9 years elementary school | 1.39 | 0.9 | 2.14 |  |
| Education, Three years high school or less than 3 years in college/university | 1.3 | 1.02 | 1.66 |  |
| Education, Three years or more college or university | 1.14 | 0.94 | 1.39 |  |
|  |  |  |  |  |
| ICU patients are patients admitted to ICU with a discharge diagnosis of Covid-19 and population controls are control individuals not admitted to hospital with Covid-19 during study period. *P-value is for the non-linear representation of the continuous variable, not the difference between quartiles. *Covid-19* Corona virus disease 2019 *OR* Odds ratio *CI* Confidence interval, *ICU* Intensive care unit, *CCI* Updated Charlson comorbidity index(1, 2) | | | | |

| **Table S13. Sensitivity analysis.** Binary logistic regression on odds ratio for being on sick leave one year after inclusion. *ICU patients and hospital patients.* Only ICU patients with invasive mechanical ventilation. | | | | |
| --- | --- | --- | --- | --- |
|  |  | 95% CI | |  |
|  | OR | Lower | Upper | *P*-value |
| Age | 1.12 | 0.82 | 1.53 | 0.89* |
| Income one year before inclusion | 0.86 | 0.66 | 1.12 | <0.001* |
| Group, ICU patients (ref: hospital patients) | 5.03 | 3.89 | 6.51 | <0.001 |
| CCI (ref 0) |  |  |  | 0.002 |
| CCI 1 | 1.54 | 1.16 | 2.04 |  |
| CCI 2 | 1.3 | 0.82 | 2.07 |  |
| CCI 3 | 1.99 | 0.92 | 4.32 |  |
| CCI 4 | 2.69 | 0.97 | 7.46 |  |
| CCI 5 | 3.6 | 0.85 | 15.15 |  |
| CCI 6-11 | 2.97 | 1.11 | 7.96 |  |
| Sex, female (ref: male) | 1.78 | 1.41 | 2.24 | <0.001 |
| Civil status (ref Married) |  |  |  | <0.97 |
| Civil status, Divorced | 1.02 | 0.76 | 1.38 |  |
| Civil status, Unmarried | 0.94 | 0.72 | 1.24 |  |
| Civil status, Widow | <0.001 | <0.001 | Inf |  |
| Origin (ref Swedish) |  |  |  | <0.001 |
| Origin, Parents from high income country | 1.03 | 0.77 | 1.38 |  |
| Origin, Parents from low income country | 0.6 | 0.45 | 0.8 |  |
| Education (ref: 9 years in elementary school to less than 3 years in high school) |  |  |  | 0.53 |
| Education, less than 9 years elementary school | 0.78 | 0.48 | 1.25 |  |
| Education, Three years high school or less than 3 years in college/university | 0.89 | 0.64 | 1.22 |  |
| Education, Three years or more college or university | 1.05 | 0.81 | 1.35 |  |
|  |  |  |  |  |
| ICU patients are patients admitted to ICU with a discharge diagnosis of Covid-19 and hospital patients are patients admitted to hospital, but not ICU, with a Covid-19 discharge diagnosis. *P-value is for the non-linear representation of the continuous variable, not the difference between quartiles. *Covid-19* Corona virus disease 2019 *OR* Odds ratio *CI* Confidence interval, *ICU* Intensive care unit, *CCI* Updated Charlson comorbidity index(1, 2) | | | | |

| **Table S14. Sensitivity analysis.** Binary logistic regression on odds ratio for being on sick leave one year after inclusion. *ICU patients and population controls.* Only ICU patients with invasive mechanical ventilation. | | | | |
| --- | --- | --- | --- | --- |
|  |  | 95% CI | |  |
|  | OR | Lower | Upper | *P*-value |
| Age | 1.22 | 0.68 | 1.82 | 0.55* |
| Group, ICU patients (ref: population controls) | 9.02 | 0.67 | 2.25 | <0.001 |
| CCI (ref 0) |  | 0.56 | 4.62 | 0.19 |
| CCI 1 | 1.11 | 0.46 | 7.19 |  |
| CCI 2 | 1.23 | 1.51 | 47.18 |  |
| CCI 3 | 1.61 | 0.52 | 16.28 |  |
| CCI 4 | 1.82 | 1.2 | 2.25 |  |
| CCI 5 | 8.44 | 0.68 | 1.82 |  |
| CCI 6-11 | 2.92 | 0.67 | 2.25 |  |
| Sex, female (ref: male) | 1.65 | 0.56 | 4.62 | 0.002 |
| Civil status (ref Married) |  |  |  | 0.27 |
| Civil status, Divorced | 1.01 | 0.68 | 1.5 |  |
| Civil status, Unmarried | 0.7 | 0.48 | 1.02 |  |
| Civil status, Widow | 0.55 | 0.07 | 4.5 |  |
| Origin (ref Swedish) |  |  |  | 0.12 |
| Origin, Parents from high income country | 1.5 | 1.02 | 2.22 |  |
| Origin, Parents from low income country | 1.11 | 0.76 | 1.63 |  |
| Education (ref: 9 years in elementary school to less than 3 years in high school) |  |  |  | 0.006 |
| Education, less than 9 years elementary school | 0.52 | 0.26 | 1.03 |  |
| Education, Three years high school or less than 3 years in college/university | 0.49 | 0.32 | 0.75 |  |
| Education, Three years or more college or university | 0.82 | 0.59 | 1.15 |  |
|  |  |  |  |  |
| ICU patients are patients admitted to ICU with a discharge diagnosis of Covid-19 and population controls are control individuals not admitted to hospital with Covid-19 during study period. *P-value is for the non-linear representation of the continuous variable, not the difference between quartiles. *Covid-19* Corona virus disease 2019 *OR* Odds ratio *CI* Confidence interval, *ICU* Intensive care unit, *CCI* Updated Charlson comorbidity index(1, 2) | | | | |

| **Table S15. Sensitivity analysis.** Ordinary least squares linear regression on the number of sick leave free days alive the first year after inclusion. *ICU patients and hospital patients.* | | | | |
| --- | --- | --- | --- | --- |
|  |  | 95% CI | |  |
|  | Beta | Lower | Upper | *P*-value |
| Age | -18.57 | -24.56 | -12.57 | 0.27* |
| Income one year before inclusion | -2.35 | -7.13 | 2.43 | <0.001* |
| Group, ICU patients (ref: hospital patients) | -110.38 | -116.18 | -104.58 | <0.001 |
| CCI (ref 0) |  |  |  | <0.001 |
| CCI 1 | -16.34 | -22.52 | -10.16 |  |
| CCI 2 | -30.03 | -39.55 | -20.51 |  |
| CCI 3 | -32.46 | -50.06 | -14.86 |  |
| CCI 4 | -73.21 | -97.32 | -49.10 |  |
| CCI 5 | -71.97 | -113.49 | -30.45 |  |
| CCI 6-11 | -98.06 | -120.24 | -75.88 |  |
| Sex, female (ref: male) | -9.38 | -13.88 | -4.88 | <0.001 |
| Civil status (ref Married) |  |  |  | 0.70 |
| Civil status, Divorced | 0.20 | -5.70 | 6.09 |  |
| Civil status, Unmarried | -1.01 | -6.46 | 4.44 |  |
| Civil status, Widow | 12.31 | -9.16 | 33.77 |  |
| Origin (ref Swedish) |  |  |  | 0.001 |
| Origin, Parents from high income country | -5.54 | -11.63 | 0.54 |  |
| Origin, Parents from low income country | 5.65 | 0.33 | 10.97 |  |
| Education (ref: 9 years in elementary school to less than 3 years in high school) |  |  |  | 0.57 |
| Education, less than 9 years elementary school | 4.14 | -3.89 | 12.17 |  |
| Education, Three years high school or less than 3 years in college/university | 3.01 | -3.12 | 9.13 |  |
| Education, Three years or more college or university | -0.02 | -5.22 | 5.17 |  |
|  |  |  |  |  |
| ICU patients are patients admitted to ICU with a discharge diagnosis of Covid-19 and hospital patients are patients admitted to hospital, but not ICU, with a Covid-19 discharge diagnosis. *P-value is for the non-linear representation of the continuous variable, not the difference between quartiles. *Covid-19* Corona virus disease 2019 *OR* Odds ratio *CI* Confidence interval, *ICU* Intensive care unit, *CCI* Updated Charlson comorbidity index(1, 2) | | | | |

| **Table S16. Sensitivity analysis.** Ordinary least squares linear regression on the number of sick leave free days alive the first year after inclusion. *ICU patients and population controls.* | | | | |
| --- | --- | --- | --- | --- |
|  |  | 95% CI | |  |
|  | Beta | Lower | Upper | *P*-value |
| Age | -13.44 | -19.29 | -7.58 | 0.009* |
| Income one year before inclusion | -1.39 | -6.00 | 3.22 | <0.001* |
| Group, ICU patients (ref: population controls) | -137.08 | -142.32 | -131.84 | <0.001 |
| CCI (ref 0) |  |  |  | <0.001 |
| CCI 1 | -17.57 | -25.91 | -9.24 |  |
| CCI 2 | -20.21 | -29.45 | -10.97 |  |
| CCI 3 | -36.03 | -55.67 | -16.38 |  |
| CCI 4 | -60.64 | -88.99 | -32.29 |  |
| CCI 5 | -51.66 | -96.93 | -6.40 |  |
| CCI 6-11 | -34.99 | -68.98 | -1.00 |  |
| Sex, female (ref: male) | -1.25 | -5.61 | 3.12 | 0.58 |
| Civil status (ref Married) |  |  |  | 0.71 |
| Civil status, Divorced | -3.23 | -8.69 | 2.23 |  |
| Civil status, Unmarried | -1.09 | -5.59 | 3.40 |  |
| Civil status, Widow | -1.85 | -26.85 | 23.15 |  |
| Origin (ref Swedish) |  |  |  | <0.001 |
| Origin, Parents from high income country | -13.56 | -19.08 | -8.05 |  |
| Origin, Parents from low income country | -2.80 | -8.36 | 2.76 |  |
| Education (ref: 9 years in elementary school to less than 3 years in high school) |  |  |  | 0.39 |
| Education, less than 9 years elementary school | 3.39 | -7.18 | 13.96 |  |
| Education, Three years high school or less than 3 years in college/university | 4.02 | -1.28 | 9.31 |  |
| Education, Three years or more college or university | 0.11 | -4.46 | 4.69 |  |
|  |  |  |  |  |
| ICU patients are patients admitted to ICU with a discharge diagnosis of Covid-19 and population controls are control individuals not admitted to hospital with Covid-19 during study period. *P-value is for the non-linear representation of the continuous variable, not the difference between quartiles. *Covid-19* Corona virus disease 2019 *OR* Odds ratio *CI* Confidence interval, *ICU* Intensive care unit, *CCI* Updated Charlson comorbidity index(1, 2) | | | | |

| **Table S17. Sensitivity analysis.** Ordinal logistic regression on odds ratio for having one or several more sick leave free days. *ICU patients and hospital patients.* Comorbid mood and anxiety disorders were added to the model. | | | | |
| --- | --- | --- | --- | --- |
|  |  | 95% CI | |  |
|  | OR | Lower | Upper | *P*-value |
| Age | 0.63 | 0.55 | 0.72 | 0.044* |
| Income one year before inclusion | 0.62 | 0.56 | 0.70 | <0.001* |
| Group, ICU patients (ref: hospital patients) | 0.18 | 0.15 | 0.20 | <0.001 |
| CCI (ref 0) |  |  |  | 0.004 |
| CCI 1 | 0.76 | 0.66 | 0.88 |  |
| CCI 2 | 0.85 | 0.68 | 1.07 |  |
| CCI 3 | 0.75 | 0.49 | 1.17 |  |
| CCI 4 | 0.56 | 0.29 | 1.09 |  |
| CCI 5 | 0.88 | 0.27 | 2.91 |  |
| CCI 6-11 | 0.87 | 0.44 | 1.71 |  |
| Sex, female (ref: male) | 0.78 | 0.71 | 0.87 | <0.001 |
| Civil status (ref Married) |  |  |  | 0.31 |
| Civil status, Divorced | 1.03 | 0.90 | 1.17 |  |
| Civil status, Unmarried | 1.00 | 0.88 | 1.13 |  |
| Civil status, Widow | 1.74 | 0.98 | 3.11 |  |
| Origin (ref Swedish) |  |  |  | <0.001 |
| Origin, Parents from high income country | 0.85 | 0.74 | 0.97 |  |
| Origin, Parents from low income country | 1.15 | 1.02 | 1.29 |  |
| Education (ref: 9 years in elementary school to less than 3 years in high school) |  |  |  | 0.005 |
| Education, less than 9 years elementary school | 1.29 | 1.06 | 1.58 |  |
| Education, Three years high school or less than 3 years in college/university | 1.16 | 1.01 | 1.33 |  |
| Education, Three years or more college or university | 0.97 | 0.87 | 1.09 |  |
| Mood and anxiety disorders | 1.29 | 1.06 | 1.58 | <0.001 |
| ICU patients are patients admitted to ICU with a discharge diagnosis of Covid-19 and hospital patients are patients admitted to hospital, but not ICU, with a Covid-19 discharge. *P-value is for the non-linear representation of the continuous variable, not the difference between quartiles. *Covid-19* Corona virus disease 2019 *OR* Odds ratio *CI* Confidence interval, *ICU* Intensive care unit, *CCI* Updated Charlson comorbidity index(1, 2) | | | | |

| **Table S18. Sensitivity analysis.** Ordinal logistic regression on odds ratio for having one or several more sick leave free days*. Covid-19 ICU patients and population controls.* Comorbid mood and anxiety disorders were added to the model. | | | | |
| --- | --- | --- | --- | --- |
|  |  | 95% CI | |  |
|  | OR | Lower | Upper | *P*-value |
| Age | 0.65 | 0.51 | 0.84 | 0.082* |
| Income one year before inclusion | 0.90 | 0.74 | 1.10 | <0.001* |
| Group, ICU patients (ref: population controls) | 0.03 | 0.02 | 0.03 | <0.001 |
| CCI (ref 0) |  |  |  | 0.021 |
| CCI 1 | 0.83 | 0.62 | 1.09 |  |
| CCI 2 | 0.68 | 0.48 | 0.95 |  |
| CCI 3 | 0.77 | 0.4 | 1.48 |  |
| CCI 4 | 0.29 | 0.12 | 0.69 |  |
| CCI 5 | 0.39 | 0.09 | 1.65 |  |
| CCI 6-11 | 0.89 | 0.21 | 3.80 |  |
| Sex, female (ref: male) | 0.78 | 0.65 | 0.93 | 0.007 |
| Civil status (ref Married) |  |  |  | 0.34 |
| Civil status, Divorced | 0.89 | 0.71 | 1.11 |  |
| Civil status, Unmarried | 1.12 | 0.93 | 1.36 |  |
| Civil status, Widow | 1.22 | 0.46 | 3.23 |  |
| Origin (ref Swedish) |  |  |  | 0.002 |
| Origin, Parents from high income country | 0.70 | 0.56 | 0.87 |  |
| Origin, Parents from low income country | 1.03 | 0.82 | 1.28 |  |
| Education (ref: 9 years in elementary school to less than 3 years in high school) |  |  |  | 0.009 |
| Education, less than 9 years elementary school | 1.61 | 1.05 | 2.47 |  |
| Education, Three years high school or less than 3 years in college/university | 1.40 | 1.11 | 1.76 |  |
| Education, Three years or more college or university | 1.21 | 1.01 | 1.46 |  |
| Mood and anxiety disorders | 0.66 | 0.48 | 0.89 | 0.006 |
| ICU patients are patients admitted to ICU with a discharge diagnosis of Covid-19 and population controls are control individuals not admitted to hospital with Covid-19 during study period. *P-value is for the non-linear representation of the continuous variable, not the difference between quartiles. *Covid-19* Corona virus disease 2019 *OR* Odds ratio *CI* Confidence interval, *ICU* Intensive care unit, *CCI* Updated Charlson comorbidity index(1, 2) | | | | |

| **Table S19. Sensitivity analysis.** Binary logistic regression on odds ratio for being on sick leave one year after inclusion. *ICU patients and hospital patients.* Comorbid mood and anxiety disorders were added to the model. | | | | |
| --- | --- | --- | --- | --- |
|  |  | 95% CI | |  |
|  | OR | Lower | Upper | *P*-value |
| Age | 1.16 | 0.85 | 1.57 | 0.93* |
| Income one year before inclusion | 0.92 | 0.71 | 1.18 | <0.001* |
| Group, ICU patients (ref: hospital patients) | 3.61 | 2.85 | 4.57 | <0.001 |
| CCI (ref 0) |  |  |  | 0.002 |
| CCI 1 | 1.57 | 1.21 | 2.06 |  |
| CCI 2 | 1.50 | 0.98 | 2.31 |  |
| CCI 3 | 1.84 | 0.90 | 3.78 |  |
| CCI 4 | 2.61 | 0.95 | 7.18 |  |
| CCI 5 | 2.99 | 0.74 | 12.13 |  |
| CCI 6-11 | 2.69 | 1.01 | 7.14 |  |
| Sex, female (ref: male) | 1.79 | 1.43 | 2.24 |  |
| Civil status (ref Married) |  |  |  | 0.48 |
| Civil status, Divorced | 0.98 | 0.74 | 1.32 |  |
| Civil status, Unmarried | 0.95 | 0.73 | 1.24 |  |
| Civil status, Widow | 0.21 | 0.03 | 1.55 |  |
| Origin (ref Swedish) |  |  |  | 0.001 |
| Origin, Parents from high income country | 1.07 | 0.81 | 1.42 |  |
| Origin, Parents from low income country | 0.63 | 0.48 | 0.83 |  |
| Education (ref: 9 years in elementary school to less than 3 years in high school) |  |  |  | 0.43 |
| Education, less than 9 years elementary school | 0.75 | 0.47 | 1.21 |  |
| Education, Three years high school or less than 3 years in college/university | 0.84 | 0.62 | 1.14 |  |
| Education, Three years or more college or university | 1.00 | 0.78 | 1.29 |  |
| Mood and anxiety disorders | 1.95 | 1.45 | 2.63 |  |
| ICU patients are patients admitted to ICU with a discharge diagnosis of Covid-19 and hospital patients are patients admitted to hospital, but not ICU, with a Covid-19 discharge. *P-value is for the non-linear representation of the continuous variable, not the difference between quartiles. *Covid-19* Corona virus disease 2019 *OR* Odds ratio *CI* Confidence interval, *ICU* Intensive care unit, *CCI* Updated Charlson comorbidity index(1, 2) | | | | |

| **Table S20. Sensitivity analysis.** Binary logistic regression on odds ratio for being on sick leave one year after inclusion. *ICU patients and population controls.* Comorbid mood and anxiety disorders were added to the model. | | | | |
| --- | --- | --- | --- | --- |
|  |  | 95% CI | |  |
|  | OR | Lower | Upper | *P*-value |
| Age | 1.28 | 0.83 | 1.97 | 0.38* |
| Group, ICU patients (ref: population controls) | 6.36 | 4.70 | 8.60 | <0.001 |
| CCI (ref 0) |  |  |  | 0.11 |
| CCI 1 | 1.35 | 0.88 | 2.06 |  |
| CCI 2 | 1.54 | 0.90 | 2.65 |  |
| CCI 3 | 1.51 | 0.60 | 3.82 |  |
| CCI 4 | 1.83 | 0.47 | 7.11 |  |
| CCI 5 | 6.73 | 1.35 | 33.57 |  |
| CCI 6-11 | 2.55 | 0.51 | 12.85 |  |
| Sex, female (ref: male) | 1.74 | 1.3 | 2.34 | <0.001 |
| Civil status (ref Married) |  |  |  | 0.39 |
| Civil status, Divorced | 0.99 | 0.68 | 1.44 |  |
| Civil status, Unmarried | 0.74 | 0.52 | 1.05 |  |
| Civil status, Widow | 0.80 | 0.17 | 3.67 |  |
| Origin (ref Swedish) |  |  |  | 0.084 |
| Origin, Parents from high income country | 1.50 | 1.04 | 2.17 |  |
| Origin, Parents from low income country | 1.06 | 0.74 | 1.51 |  |
| Education (ref: 9 years in elementary school to less than 3 years in high school) |  |  |  | 0.002 |
| Education, less than 9 years elementary school | 0.51 | 0.26 | 0.99 |  |
| Education, Three years high school or less than 3 years in college/university | 0.48 | 0.32 | 0.72 |  |
| Education, Three years or more college or university | 0.80 | 0.58 | 1.09 |  |
| Mood and anxiety disorders | 1.88 | 1.23 | 2.88 | 0.004 |
| ICU patients are patients admitted to ICU with a discharge diagnosis of Covid-19 and hospital patients are patients admitted to hospital, but not ICU, with a Covid-19 discharge. *P-value is for the non-linear representation of the continuous variable, not the difference between quartiles. *Covid-19* Corona virus disease 2019 *OR* Odds ratio *CI* Confidence interval, *ICU* Intensive care unit, *CCI* Updated Charlson comorbidity index(1, 2) | | | | |

| **Table S21. Sensitivity analysis.** Ordinal logistic regression on odds ratio for having one or several more sick leave free days. *ICU patients and hospital patients.* Patients with extensive sick leave before inclusion are included in the cohort and the preceding number of sick leave days is adjusted for. | | | | |
| --- | --- | --- | --- | --- |
|  |  | 95% CI | |  |
|  | OR | Lower | Upper | *P*-value |
| Age | 0.6 | 0.53 | 0.68 | 0.005* |
| Income one year before inclusion | 0.75 | 0.66 | 0.84 | <0.001* |
| Group, ICU patients (ref: hospital patients) | 0.16 | 0.14 | 0.18 | <0.001 |
| CCI (ref 0) |  |  |  | <0.001 |
| CCI 1 | 0.73 | 0.64 | 0.83 |  |
| CCI 2 | 0.65 | 0.54 | 0.78 |  |
| CCI 3 | 0.62 | 0.43 | 0.89 |  |
| CCI 4 | 0.41 | 0.25 | 0.68 |  |
| CCI 5 | 0.53 | 0.23 | 1.23 |  |
| CCI 6-11 | 0.26 | 0.16 | 0.41 |  |
| Sex, female (ref: male) | 0.86 | 0.79 | 0.95 | 0.002 |
| Civil status (ref Married) |  |  |  | 0.13 |
| Civil status, Divorced | 1.03 | 0.92 | 1.17 |  |
| Civil status, Unmarried | 0.92 | 0.82 | 1.02 |  |
| Civil status, Widow | 1.47 | 0.89 | 2.43 |  |
| Origin (ref Swedish) |  |  |  | <0.001 |
| Origin, Parents from high income country | 0.88 | 0.78 | 1 |  |
| Origin, Parents from low income country | 1.13 | 1.01 | 1.26 |  |
| Education (ref: 9 years in elementary school to less than 3 years in high school) |  |  |  | 0.006 |
| Education, less than 9 years elementary school | 1.15 | 0.96 | 1.38 |  |
| Education, Three years high school or less than 3 years in college/university | 1.12 | 0.98 | 1.27 |  |
| Education, Three years or more college or university | 0.92 | 0.83 | 1.02 |  |
| Sick leave days 6 months to 2 weeks before admission | 0.01 | <0.01 | 0.01 | <0.001* |
| ICU patients are patients admitted to ICU with a discharge diagnosis of Covid-19 and hospital patients are patients admitted to hospital, but not ICU, with a Covid-19 discharge. *P-value is for the non-linear representation of the continuous variable, not the difference between quartiles. *Covid-19* Corona virus disease 2019 *OR* Odds ratio *CI* Confidence interval, *ICU* Intensive care unit, *CCI* Updated Charlson comorbidity index(1, 2) | | | | |

| **Table S22. Sensitivity analysis.** Ordinal logistic regression on odds ratio for having one or several more sick leave free days*. Covid-19 ICU patients and population controls.* Patients with extensive sick leave before inclusion are included in the cohort and the preceding number of sick leave days is adjusted for. | | | | |
| --- | --- | --- | --- | --- |
|  |  | 95% CI | |  |
|  | OR | Lower | Upper | *P*-value |
| Age | 0.58 | 0.47 | 0.73 | 0.01* |
| Income one year before inclusion | 1.13 | 0.94 | 1.37 | <0.001* |
| Group, ICU patients (ref: population controls) | 0.03 | 0.02 | 0.03 | <0.001 |
| CCI (ref 0) |  |  |  | <0.001 |
| CCI 1 | 0.86 | 0.67 | 1.09 |  |
| CCI 2 | 0.62 | 0.47 | 0.84 |  |
| CCI 3 | 0.55 | 0.33 | 0.93 |  |
| CCI 4 | 0.29 | 0.13 | 0.62 |  |
| CCI 5 | 0.73 | 0.25 | 2.18 |  |
| CCI 6-11 | 0.36 | 0.15 | 0.87 |  |
| Sex, female (ref: male) | 0.9 | 0.76 | 1.05 | 0.18 |
| Civil status (ref Married) |  |  |  | 0.36 |
| Civil status, Divorced | 0.91 | 0.75 | 1.1 |  |
| Civil status, Unmarried | 1.02 | 0.86 | 1.21 |  |
| Civil status, Widow | 0.56 | 0.26 | 1.22 |  |
| Origin (ref Swedish) |  |  |  | 0.002 |
| Origin, Parents from high income country | 0.7 | 0.58 | 0.86 |  |
| Origin, Parents from low income country | 0.97 | 0.79 | 1.18 |  |
| Education (ref: 9 years in elementary school to less than 3 years in high school) |  |  |  | 0.19 |
| Education, less than 9 years elementary school | 1.11 | 0.77 | 1.61 |  |
| Education, Three years high school or less than 3 years in college/university | 1.24 | 1.01 | 1.52 |  |
| Education, Three years or more college or university | 1.01 | 0.86 | 1.2 |  |
| Sick leave days 6 months to 2 weeks before admission | <0.01 | <0.01 | <0.01 | <0.001* |
| ICU patients are patients admitted to ICU with a discharge diagnosis of Covid-19 and population controls are control individuals not admitted to hospital with Covid-19 during study period. *P-value is for the non-linear representation of the continuous variable, not the difference between quartiles. *Covid-19* Corona virus disease 2019 *OR* Odds ratio *CI* Confidence interval, *ICU* Intensive care unit, *CCI* Updated Charlson comorbidity index(1, 2) | | | | |

| **Table S23. Sensitivity analysis.** Binary logistic regression on odds ratio for being on sick leave one year after inclusion. *ICU patients and hospital patients.* Patients with extensive sick leave before inclusion are included in the cohort and the preceding number of sick leave days is adjusted for. | | | | |
| --- | --- | --- | --- | --- |
|  |  | 95% CI | |  |
|  | OR | Lower | Upper | *P*-value |
| Age | 1.32 | 1.03 | 1.7 | 0.53* |
| Income one year before inclusion | 0.57 | 0.46 | 0.7 | <0.001* |
| Group, ICU patients (ref: hospital patients) | 3.04 | 2.45 | 3.76 | <0.001 |
| CCI (ref 0) |  |  |  | <0.001 |
| CCI 1 | 1.66 | 1.33 | 2.09 |  |
| CCI 2 | 1.55 | 1.12 | 2.14 |  |
| CCI 3 | 2.51 | 1.48 | 4.25 |  |
| CCI 4 | 1.86 | 0.84 | 4.12 |  |
| CCI 5 | 2.51 | 0.8 | 7.82 |  |
| CCI 6-11 | 1.93 | 1 | 3.72 |  |
| Sex, female (ref: male) | 1.58 | 1.31 | 1.9 | <0.001 |
| Civil status (ref Married) |  |  |  | 0.19 |
| Civil status, Divorced | 0.97 | 0.76 | 1.22 |  |
| Civil status, Unmarried | 1.09 | 0.87 | 1.35 |  |
| Civil status, Widow | 0.28 | 0.08 | 0.99 |  |
| Origin (ref Swedish) |  |  |  | <0.001 |
| Origin, Parents from high income country | 0.97 | 0.76 | 1.22 |  |
| Origin, Parents from low income country | 0.65 | 0.52 | 0.81 |  |
| Education (ref: 9 years in elementary school to less than 3 years in high school) |  |  |  | 0.68 |
| Education, less than 9 years elementary school | 0.91 | 0.64 | 1.28 |  |
| Education, Three years high school or less than 3 years in college/university | 0.92 | 0.7 | 1.19 |  |
| Education, Three years or more college or university | 1.06 | 0.86 | 1.3 |  |
| Sick leave days 6 months to 2 weeks before admission | 83.61 | 64.74 | 107.99 | 0.001* |
| ICU patients are patients admitted to ICU with a discharge diagnosis of Covid-19 and hospital patients are patients admitted to hospital, but not ICU, with a Covid-19 discharge. *P-value is for the non-linear representation of the continuous variable, not the difference between quartiles. *Covid-19* Corona virus disease 2019 *OR* Odds ratio *CI* Confidence interval, *ICU* Intensive care unit, *CCI* Updated Charlson comorbidity index(1, 2) | | | | |

| **Table S24. Sensitivity analysis.** Binary logistic regression on odds ratio for being on sick leave one year after inclusion. *ICU patients and population controls.* Patients with extensive sick leave before inclusion are included in the cohort and the preceding number of sick leave days is adjusted for. | | | | |
| --- | --- | --- | --- | --- |
|  |  | 95% CI | |  |
|  | OR | Lower | Upper | *P*-value |
| Age | 1.45 | 1 | 2.12 | 0.065* |
| Group, ICU patients (ref: population controls) | 5.5 | 4.18 | 7.24 | <0.001 |
| CCI (ref 0) |  |  |  | 0.36 |
| CCI 1 | 1.23 | 0.83 | 1.81 |  |
| CCI 2 | 1.25 | 0.78 | 1.99 |  |
| CCI 3 | 2.04 | 0.98 | 4.25 |  |
| CCI 4 | 1.51 | 0.42 | 5.35 |  |
| CCI 5 | 2.43 | 0.56 | 10.6 |  |
| CCI 6-11 | 1.71 | 0.46 | 6.38 |  |
| Sex, female (ref: male) | 1.48 | 1.14 | 1.91 | 0.003 |
| Civil status (ref Married) |  |  |  | 0.99 |
| Civil status, Divorced | 1.01 | 0.73 | 1.39 |  |
| Civil status, Unmarried | 0.85 | 0.63 | 1.14 |  |
| Civil status, Widow | 1.51 | 0.46 | 4.96 |  |
| Origin (ref Swedish) |  |  |  | 0.04 |
| Origin, Parents from high income country | 1.51 | 1.09 | 2.07 |  |
| Origin, Parents from low income country | 1.12 | 0.81 | 1.54 |  |
| Education (ref: 9 years in elementary school to less than 3 years in high school) |  |  |  | <0.001 |
| Education, less than 9 years elementary school | 0.64 | 0.36 | 1.14 |  |
| Education, Three years high school or less than 3 years in college/university | 0.46 | 0.32 | 0.65 |  |
| Education, Three years or more college or university | 0.85 | 0.65 | 1.12 |  |
| Sick leave days 6 months to 2 weeks before admission | 215.9 | 153.18 | 304.31 | <0.001* |
| ICU patients are patients admitted to ICU with a discharge diagnosis of Covid-19 and hospital patients are patients admitted to hospital, but not ICU, with a Covid-19 discharge. *P-value is for the non-linear representation of the continuous variable, not the difference between quartiles. *Covid-19* Corona virus disease 2019 *OR* Odds ratio *CI* Confidence interval, *ICU* Intensive care unit, *CCI* Updated Charlson comorbidity index(1, 2) | | | | |

## **References**

1. Charlson ME, Pompei P, Ales KL, MacKenzie CR. A new method of classifying prognostic comorbidity in longitudinal studies: Development and validation. Journal of Chronic Diseases. 1987;40(5):373-83.

2. Quan H, Li B, Couris CM, Fushimi K, Graham P, Hider P, et al. Updating and validating the Charlson comorbidity index and score for risk adjustment in hospital discharge abstracts using data from 6 countries. Am J Epidemiol. 2011;173(6):676-82.

3. International Standard Classification of Education (ISCED): International Labour Organization; 2014 [cited 2024 13 December 2024]. Available from: 13 December 2024.

4. Moreno RP, Metnitz PG, Almeida E, Jordan B, Bauer P, Campos RA, et al. SAPS 3--From evaluation of the patient to evaluation of the intensive care unit. Part 2: Development of a prognostic model for hospital mortality at ICU admission. Intensive Care Med. 2005;31(10):1345-55.

5. Exchange-Rates.org: MBH Media, Inc.; [Available from: <https://www.exchange-rates.org/Rate/SEK/USD/12-31-2019>.
